# PP2A Promotes the Symmetric Division of MUC1‐Dominant Cancer Stem‐Like Cells in Small Cell Lung Cancer

**DOI:** 10.1002/advs.202503545

**Published:** 2025-05-11

**Authors:** Shengze Li, Xinran Dong, Kexing Gao, Yiyang Wang, Min Li, Huayun Deng, Shuangyu Ma, Yaping Lv, Wei Jin, Quanfu Li, Yuming Wang, Xiaodong Liao, Kangjing Bian, Aiwu Zhou, Chengping Hu, Lei Huang

**Affiliations:** ^1^ Department of Histoembryology Genetics and Developmental Biology Key Laboratory of Cell Differentiation and Apoptosis of Chinese Ministry of Education Shanghai Key Laboratory of Reproductive Medicine Shanghai Jiao Tong University School of Medicine Shanghai 200025 China; ^2^ Center for Molecular Medicine Children's Hospital of Fudan University Shanghai 201102 China; ^3^ Department of Thoracic Surgery Shanghai Chest Hospital Shanghai Jiao Tong University School of Medicine Shanghai 200030 China; ^4^ Department of Respiratory Medicine Xiangya Hospital Central South University Hunan 410028 China; ^5^ Department of Laboratory Medicine Ren Ji Hospital School of Medicine Shanghai Jiao Tong University Shanghai 201112 China

**Keywords:** CSLCs, MUC1, NUMB, PKCζ, PP2A, SCLC

## Abstract

Small cell lung cancer (SCLC) is the most aggressive and lethal subtype of lung cancer. Cancer stem‐like cells (CSLCs) are primarily responsible for carcinogenesis, therapeutic resistance, and tumor recurrence. This study reported that high level of mucin1 (MUC1) is associated with poor patient survival in SCLC. MUC1 expression peaks during the G2/M phase and facilitates symmetric division and expansion of CSLCs. Mechanistically, the interaction of MUC1 and protein phosphatase 2A (PP2A) results in augmented PP2A activity, which leads to reduced phosphorylation of protein kinase C ζ (PKCζ), ultimately decreases phosphorylation of NUMB. Both pharmacological and genetic strategies demonstrate that targeted‐inhibition of the MUC1‐PP2A axis pointedly rescues phosphorylation of PKCζ and NUMB, thereby shifting CSLCs towards asymmetric division and represses CSLCs proliferation. Conversely, inhibitor of PKCζ suppresses phosphorylation of NUMB, promotes symmetric division and induces enrichment of CSLCs. Moreover, combination of etoposide and inhibitors of MUC1‐PP2A pathway efficiently constrains tumor growth in vitro and in vivo. Importantly, a negative correlation is observed between MUC1 and phosphorylation of PKCζ and NUMB in SCLC patients. Therefore, this study reveals a novel mechanism by which MUC1‐PP2A awakes CSLC expansion via switching symmetric division, suggesting a potential therapeutic strategy for MUC1‐positive SCLC.

## Graphical abstract

During the G2/M phase, mucin1 (MUC1) activates protein phosphatase 2A (PP2A), leading to reduced phosphorylation of protein kinase C ζ (PKCζ) and NUMB, which promotes symmetric division and expansion of cancer stem‐like cells (CSLCs), thereby driving tumorigenesis. Combined inhibition of the MUC1–PP2A axis using LB100/GO203 with etoposide restores PKCζ activity and enhances NUMB phosphorylation, effectively suppressing CSLCs proliferation and tumor growth.

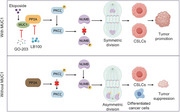

## 1 Introduction

Small cell lung cancer (SCLC) is one of the most aggressive and most lethal subtype of lung cancer, 80–85% of patients are diagnosed at the extensive stage of the disease.^[^ ^1^ ^]^ The standard treatment for extensive‐stage SCLC involves systemic chemotherapy with platinum and etoposide. SCLC is characterized by rapid cellular proliferation,^[^ ^2^ ^]^ making it highly sensitive to initial chemotherapy. However, the majority of SCLC patients experience rapid relapse, the 5 years survival rate remains below 7%.^[^ ^3^, ^4^ ^]^ One contributing factor to acquired treatment resistance is the presence of cancer stem cells (CSCs). CSCs exhibit self‐renewal capabilities and contribute to tumor invasion, metastasis, and recurrence.^[^ ^5^, ^6^, ^7^ ^]^ A CD133‐positive subpopulation has been identified in SCLC, and revealed consistent the capacity for CSCs.^[^ ^8^, ^9^, ^10^, ^11^ ^]^ Other markers, including ATP binding cassette transporter G2 (ABCG2), CD44, and sex determining region Y box protein 2 (SOX2), have also been associated with CSCs in SCLC.^[^ ^12^, ^13^, ^14^ ^]^ CSCs maintain self‐renewal through both asymmetric and symmetric divisions, which together preserve a dynamic balance in CSC populations. Asymmetric cell division is a fundamental property of stem cells, enabling them to self‐renew while producing differentiated progeny. This mechanism ensures a proper balance between stem cells and their differentiated offspring. By contrast, stem cells can shift from asymmetric to symmetric division during development or in response to injury. Such a shift often results in an expansion of stem cell populations, potentially contributing to tumorigenesis and resistance to chemotherapy and radiotherapy.^[^ ^15^, ^16^ ^]^ NUMB is a well‐known regulator of asymmetric cell division. During cell division, NUMB localizes to one side of the cell and promotes cell differentiation by inhibiting NOTCH signaling, which is critical for stem cell maintenance. The cells on the opposite side maintain their stemness, thereby completing the asymmetric division.^[^ ^15^, ^17^, ^18^ ^]^ Thus, NUMB is commonly used as a marker to distinguish stem cell division patterns.^[^ ^19^, ^20^ ^]^

Mucin1 (MUC1) is a type I transmembrane glycoprotein that forms a mucus barrier under physiological conditions, shielding epithelial cells from environmental insults.^[^ ^21^, ^22^, ^23^ ^]^ However, MUC1 is overexpressed in tumor cells, contributing to malignant transformation and tumorigenesis,^[^ ^24^, ^25^, ^26^, ^27^ ^]^ promoting tumor metastasis,^[^ ^28^ ^]^ and facilitating drug resistance and immune escape.^[^ ^29^, ^30^, ^31^, ^32^ ^]^ Our previous studies have revealed that MUC1 plays a crucial role in regulating CSCs. MUC1 promotes the enrichment of paclitaxel‐resistant CSCs by activating the epidermal growth factor receptor (EGFR) ‐ cAMP‐response element binding protein (CREB) /glucocorticoid receptor β (GRβ) ‐ interleukin 6 (IL‐6) signaling axis in cervical cancer.^[^ ^33^ ^]^ Additionally, MUC1 enhances the maintenance of breast CSCs by promoting PTEN induced putative kinase 1 (PINK1)‐dependent mitophagy via ATPase family AAA domain containing protein 3A (ATAD3A) destabilization.^[^ ^34^ ^]^ Conversely, WW domain containing E3 ubiquitin protein ligase 1 (WWP1)‐mediated degradation of MUC1 suppresses CSC self‐renewal and carcinogenesis.^[^ ^35^ ^]^

Previous studies have shown that MUC1 is highly expressed in patient‐derived SCLC cell lines, suggesting its potential as a biomarker for SCLC.^[^ ^36^ ^]^ Other group reported that miR‐543 regulates cell proliferation and migration via MUC1 in SCLC cells.^[^ ^37^ ^]^ Notably, MUC1 has been reported to promote CSC self‐renewal and tumorigenicity in SCLC by activating the myelocytomatosis viral oncogene homolog (MYC)–E2 transcription factor (E2F)–NOTCH2 signaling.^[^ ^38^ ^]^ However, the effect of MUC1 on CSC division patterns remains unclear. Here, we demonstrate that MUC1 promotes symmetric division and expansion of cancer stem‐like cells (CSLCs) in SCLC. Mechanistically, MUC1 interacts with protein phosphatase 2A (PP2A) and enhances its activity, which in turn leads to dephosphorylates protein kinase C ζ (PKCζ). This results in decreased NUMB phosphorylation, thereby shifting CSLCs toward symmetric division, promoting their expansion, and ultimately fueling tumor growth in SCLC. Importantly, targeted inhibition of either MUC1 or PP2A shifts CSLCs toward asymmetric division and suppresses tumor growth.

## 2 Results

### 2.1 High Expression of MUC1 Correlated with Poor Prognosis in SCLC

**Figure 1:**
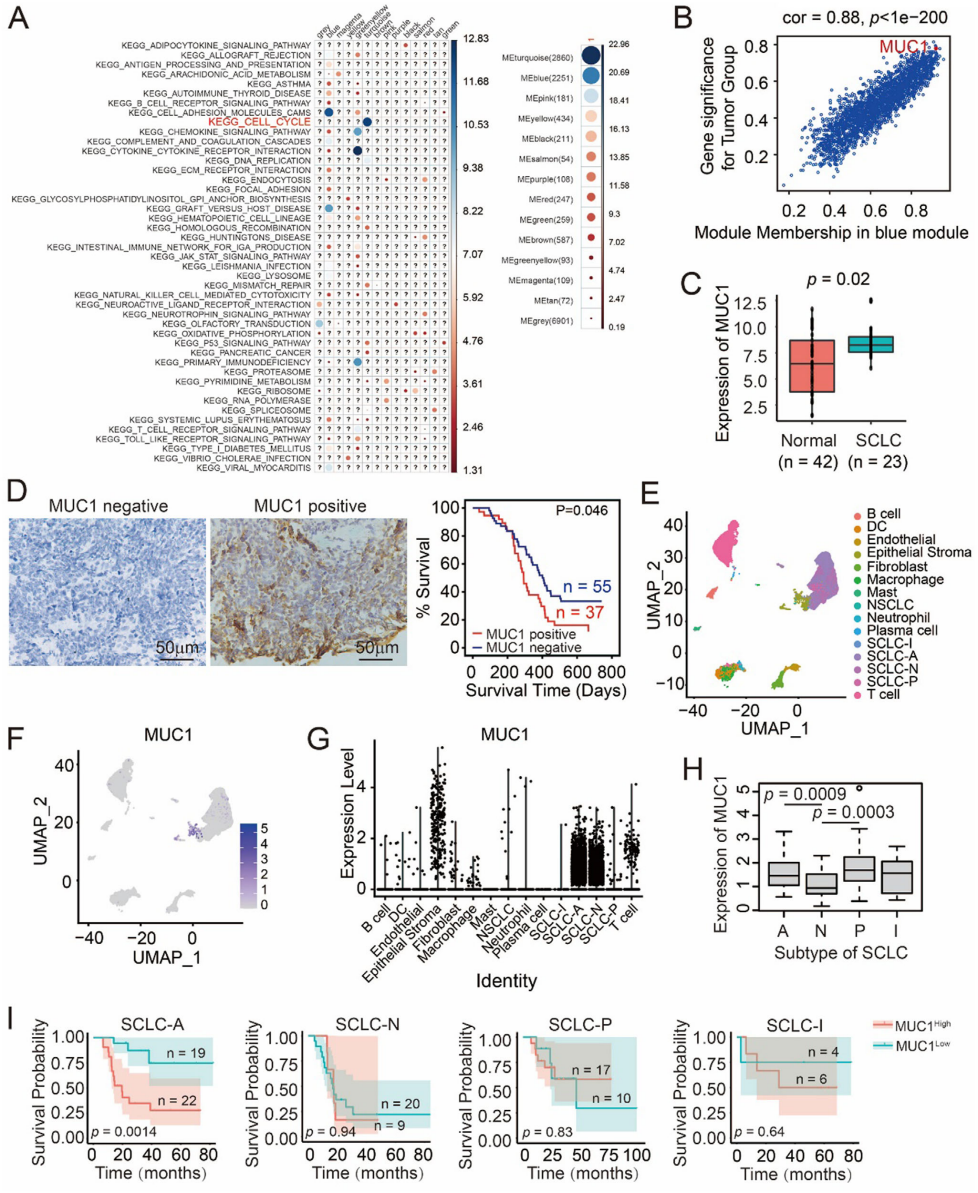
MUC1 is highly expressed in SCLC and is associated with poor patient prognosis. A) Ordering of the degree of changes in gene modules and KEGG function analysis. B) Significant differences of changes in genes in blue modules. The *y*‐axis, labeled “Gene Significance for Tumor Group,” represents the Pearson correlation coefficient between gene expression levels and the tumor status. This correlation coefficient ranges from −1 to 1, with higher positive values indicating a stronger positive correlation with the tumor status. C) Gene expression levels of *MUC1* in SCLC versus normal tissues, data from the GEO database. D) Survival time of 92 SCLC patients was negatively correlated with MUC1 expression. Patients with high levels of MUC1 are shown in red, and those with low levels of MUC1 are shown in blue. Scale bars: 50 µm. E–G) Single‐cell analysis of cell type and MUC1 expression in SCLC tumor tissues. H) MUC1 expression in four subtypes of SCLC. I) Relationship between survival time and MUC1 expression in patients with four subtypes of SCLC. Patients with high MUC1 expression are shown in red and patients with low MUC1 expression are shown in blue.

To investigate the gene expression profiles in SCLC, we analyzed transcriptome data for normal and tumor tissue of 52 small cell lung cancer patients from the GSE database (Figure S1A, Supporting Information). After normalizing 14 367 expressed genes, weighted gene coexpression network analysis identified 14 distinct gene modules based on their genetic correlation (Figure S1B,C, Supporting Information). KEGG pathway analysis of each module revealed that differentially expressed genes were mainly involved in regulating the cell cycle and cell adhesion (**Figure** **1**A). Further correlation analysis of the genes from cell cycle and cell adhesion modules revealed that *MUC1* was not only highly correlated with the gene expression levels but also with the tumor status (Figure 1B). We next compared MUC1 mRNA levels between normal and SCLC tissues using the GSE43346 dataset and found significantly elevated expression of MUC1 in SCLC tissues (Figure 1C). The survival ratio of SCLC patients was negatively correlated with MUC1 expression (Figure 1D and **Table** **1**).

**Table 1:** MUC1 expression in SCLC patients.

Features		Total	MUC1 expression	
			−	+
Sex	Female	14 (15.2%)	8 (57.1%)	6 (42.9%)
	Male	78 (84.8%)	47 (51.1%)	31 (48.9%)
Age	<60	56 (60.9%)	30 (53.6%)	26 (46.4%)
	≥60	36 (39.1%)	25 (69.4%)	11 (30.6%)
Tumor stage	Early stages I and II	4 (4.3%)	3 (75%)	1 (25%)
	Advanced stages III and IV	88 (95.7%)	52 (59.1%)	36 (40.9%)
Site	Left lung	45 (48.9%)	32 (71.1%)	13 (28.9%)
	Right lung	47 (51.1%)	23 (48.9%)	24 (51.1%)
Chemotherapy	Etoposide + cisplatin	86 (93.5%)	52 (60.5%)	34 (39.5%)
	Others	6 (6.5%)	3 (50%)	3 (50%)

Given that SCLC can be classified into four subtypes (SCLC‐A, SCLC‐N, SCLC‐P, and SCLC‐I) based on gene expression profiles.^[^ ^2^, ^39^ ^]^ To further explore MUC1 expression in SCLC subtypes, we analyzed single‐cell RNA sequencing data from tumor tissues and found that MUC1 was prominently expressed in SCLC‐A and SCLC‐N subtype cells (Figure 1E–G and **Table** **2**). Analysis of SCLC samples in the HTAN database showed relatively elevated MUC1 expression in the SCLC‐A and SCLC‐P subtypes (Figure 1H). Furthermore, survival analysis revealed that high MUC1 expression was associated with poor prognosis in SCLC‐A patients (Figure 1I). According to the expression of MUC1 in different subtypes of SCLC and the negative correlation with patient prognosis, we inferred that MUC1 is a distinguished biomarker for tumor growth and treatment of SCLC.

**Table 2:** MUC1 expression in different cell types in the single‐cell sequencing analysis.

Cell type	Number	Proportion of MUC1 expression	*p* value (vs other types)
B cell	788	0.63%	1
DC	1530	0.85%	1
Endothelial	594	1.18%	1
Epithelial stroma	1323	17.08%	5.571687 × 10^−36^
Fibroblast	800	3.13%	1
Macrophage	1051	2.38%	1
Mast	104	0.00%	1
NSCLC	220	5.00%	0.101999
Neutrophil	727	0.41%	0.999999
Plasma cell	34	0.00%	1
SCLC	20	5.00%	0.325933
SCLC‐A	31 865	11.50%	2.196489 × 10^−90^
SCLC‐N	19 279	8.59%	3.272522 × 10^−4^
SCLC‐P	3169	0.63%	1
T cell	15 639	0.67%	1

### 2.2 MUC1 Facilitates the Symmetric Division of Tumor Spheroidal Cells

**Figure 2:**
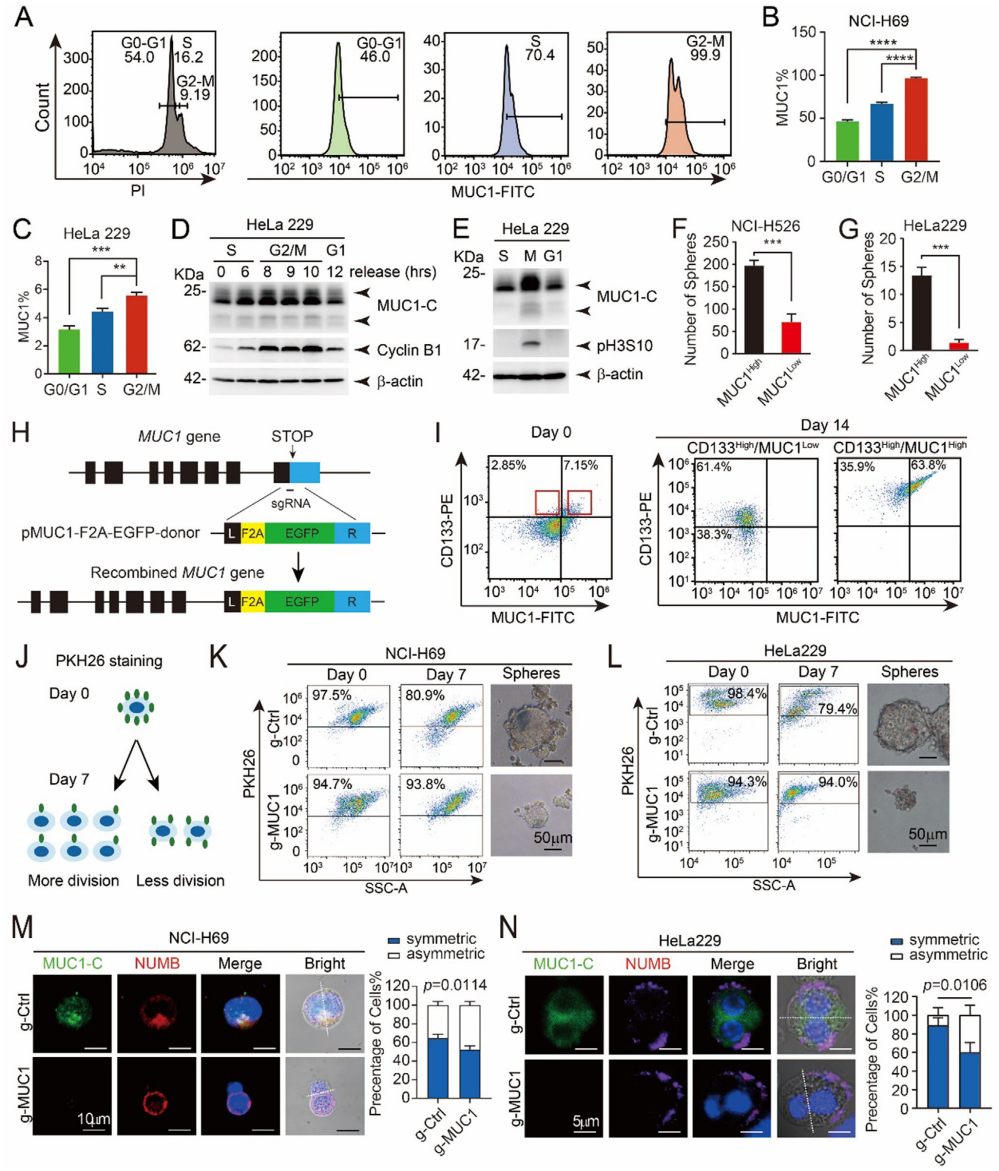
MUC1 is highly expressed in the G2/M phase and promotes symmetric division and cell proliferation. A–C) Flow cytometry analysis (FCM) of MUC1 protein levels at different cell cycle phases in NCI‐H69 (A, B) and HeLa 229 (C) cells. The data represent the mean ± standard deviation (SD) from three independent experiments. D) HeLa 229 cells were synchronized in S phase using double thymidine block, followed by release. MUC1 protein expression was assessed by western blot at various time points postrelease. Cyclin B1 was used as a marker for G2/M phase, and β‐actin served as a loading control. E) HeLa 229 cells were synchronized in S phase by double thymidine block and then arrested in M phase by treatment with nocodazole. Cells at S, M, and G1 phases were collected, and protein expression was analyzed by western blot. F,G) Evaluation of the effect of MUC1 expression on spheroid‐forming ability in NCI‐H526 (F) and HeLa 229 cells (G). The data are presented as the mean ± SD from three independent experiments. H) Schematic diagram of the EGFP‐MUC1 construct. I) FCM analysis of MUC1 expression with CD133 in NCI‐H69 cells. J) Schematic illustrating the principle of PKH26 dye labeling for tracking cell proliferation. K,L) Tumor spheroidal cells were enriched via primary sphere formation from NCI‐H69 (K) and HeLa 229 (L) cells, digested into single cells, labeled with PKH26, and analyzed by flow cytometry to determine the initial proportion of PKH26⁺ cells (day 0). Labeled cells were further cultured in sphere‐forming medium for 7 days, and the remaining PKH26⁺ population was reassessed. Scale bars: 50 µm. M,N) Sphere‐forming experiments were performed using NCI‐H69 (M) and HeLa 229 cells (N), enriched for CSLCs; spheres were digested into individual cells and continued to be cultured in sphere‐forming medium while being treated with 25 µm ovalbumin inhibitor Blebbistatin, and immunofluorescence assay was performed after 48 h. The expression and distribution of NUMB and MUC1 in the cells were observed. The proportions of symmetric and asymmetric divisions were quantified. Scale bars: 10 µm. The data are presented as the mean ± SD from three independent experiments.

We sought to determine whether MUC1 plays a role in the cell cycle. To address this question, MUC1 expression at different phases of the cell cycle was analyzed by flow cytometry in NCI‐H69 cells. The result showed significantly elevated MUC1 expression during the G2/M phase (**Figure** **2**A,B). This finding was confirmed in HeLa 229 cells which is easy to synchronize (Figure S2A, Supporting Information). We utilized thymidine double blockade to arrest cells in the S phase, followed by release and time‐course collection of cells. Western blot analysis revealed elevated MUC1 protein levels during the G2/M phase in HeLa 229 cells, with cyclin B1 serving as a positive control (Figure 2D). To confirm this, cells were synchronized in the S phase using a thymidine two‐step block, then treated with Nocodazole to arrest them in the M phase, followed by collection at the S, M, and G1 phases. Consistently, data showed that MUC1 expression peaked in the M phase (Figure 2E). Given that metaphase effectors influence cell division patterns, which are critical for CSC expansion,^[^ ^40^ ^]^ we hypothesized that MUC1 may be involved in the enrichment of tumor spheroidal cells in SCLC. Indeed, the MUC1^High^ cells showed a greater sphere‐forming capacity than MUC1^Low^ cells in both NCI‐H526 (Figure 2F and Figure S2B (Supporting Information)) and HeLa 229 cell lines (Figure 2G and Figure S2C (Supporting Information)). To assess the role of MUC1 in cell differentiation, GFP was knocked in at the C‐terminus of the *MUC1* gene using CRISPR‐Cas9 (Figure 2H). By fluorescence microscopy, we found that the CRISPR knock‐in efficiency was about 90% (Figure S2D, Supporting Information). CD133^High^ cells were isolated and further divided into MUC1^High^ and MUC1^Low^ subpopulations. After 14 days of culture in RPMI‐1640 medium with 10% FBS, the CD133^High^/MUC1^High^ cells were differentiated to CD133^High^/MUC1^Low^ cells, whereas the CD133^High^/MUC1^Low^ cells produced CD133^Low^/MUC1^Low^ cells (Figure 2I). These results indicate that high MUC1 expression significantly enhances cell stemness of CD133^High^. To assess MUC1's influence on tumor spheroidal cells proliferation, we used PKH26 dye, which dilutes with each cell division. (Figure 2J). Flow cytometry revealed that the PKH26 signal decreased more rapidly in g‐Ctrl cells than g‐MUC1 cells, indicating enhanced proliferation in MUC1‐expressing cells (Figure 2K left). Constantly, there were more and larger spheres in g‐Ctrl cells than g‐MUC1 cells (Figure 2K right). This observation was further validated in HeLa 229 cells (Figure 2L). These results suggest that MUC1 promotes the proliferation of tumor spheroidal cells. To further investigate whether MUC1 affects cell division pattern, the tumor spheroidal cells were arrested in late metaphase with oval groove inhibitor Blebbistatin and stained with NUMB. Results indicated that the proportion of symmetrical cell division was higher in g‐Ctrl cells than in g‐MUC1 cells with both NCI‐H69 (Figure 2M) and HeLa229 cell lines (Figure 2N). Taken together, these results demonstrate that MUC1 promotes the symmetric division of tumor spheroidal cells.

### 2.3 MUC1 Induces Expansion of the CSLCs in SCLC

**Figure 3:**
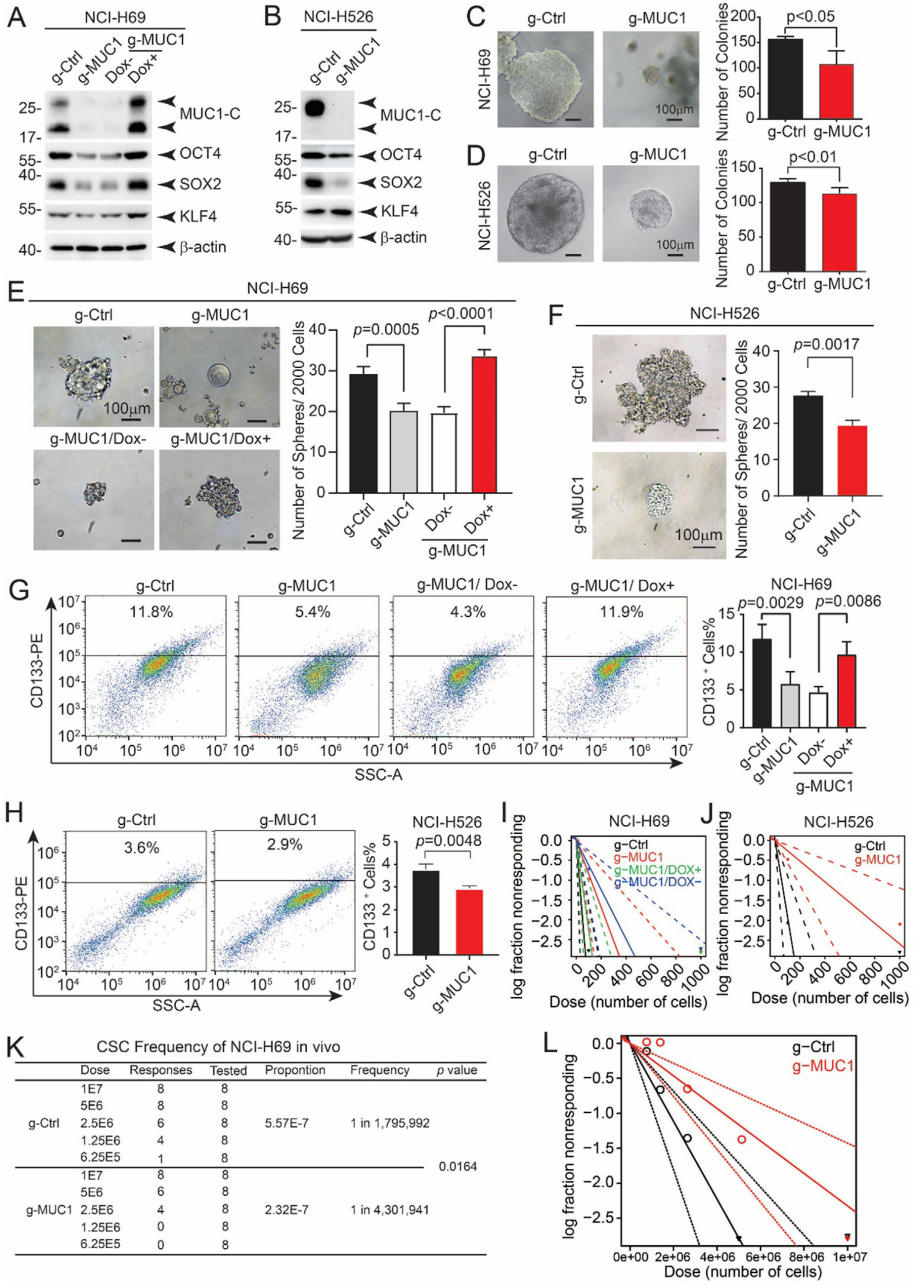
MUC1 could maintain the stemness of cancer stem‐like cells. A) Western blot analysis of stemness‐related proteins OCT4, SOX2, and KLF4 in NCI‐H69 cells before and after MUC1 knockdown and DOX‐induced re‐expression. B) Western blot analysis of OCT4, SOX2, and KLF4 in NCI‐H526 cells before and after MUC1 knockdown. C,D) Quantification of colony‐forming ability in NCI‐H69 (C) and NCI‐H526 (D) cells before and after MUC1 knockdown. Scale bars: 100 µm. The data represent the mean ± SD from three independent experiments. E) Sphere formation assay in NCI‐H69 cells with MUC1 knockout and DOX‐induced re‐expression. The number of spheres was statistically analyzed. Scale bars: 100 µm. The data represent the mean ± SD from three independent experiments. F) Sphere formation assay in NCI‐H526 cells with or without MUC1 knockout. Sphere numbers were statistically analyzed. Scale bars: 100 µm. The data represent the mean ± SD from three independent experiments. G) Flow cytometric analysis of CD133⁺ cell populations in NCI‐H69/g‐Ctrl, NCI‐H69/g‐MUC1, and NCI‐H69/g‐MUC1 cells following DOX‐induced MUC1 expression. CD133⁺ proportions were statistically analyzed. The data represent the mean ± SD from three independent experiments. H) Flow cytometric analysis of CD133⁺ cells in NCI‐H526/g‐Ctrl and NCI‐H526/g‐MUC1 cells. CD133⁺ proportions were statistically analyzed. The data represent the mean ± SD from three independent experiments. I) In vitro CSLC frequency in NCI‐H69 cells before and after MUC1 knockout, and following DOX‐induced MUC1 re‐expression in NCI‐H69/g‐MUC1 cells, determined by extreme limiting dilution analysis (ELDA). J) In vitro CSLC frequency in NCI‐H526 cells before and after MUC1 knockout determined by ELDA. K,L) In vivo CSLC frequencies in NCI‐H69 cells before and after MUC1 knockout determined by ELDA.

To investigate whether MUC1 regulates the enrichment of CSLCs in SCLC cell lines, we first examined the protein levels of stemness‐associated factors OCT4 and SOX2 in NCI‐H69 cells. The protein levels of both OCT4 and SOX2 decreased following MUC1 knockdown and were restored upon doxycycline‐induced re‐expression of MUC1 (**Figure** **3**A). Similar results were observed in the NCI‐H526 SCLC cell line (Figure 3B). Colony‐formation assays demonstrated that larger and more colonies cells showed in g‐Ctrl cells than g‐MUC1 cells with both NCI‐H69 (Figure 3C) and NCI‐H526 cell lines (Figure 3D). The number of mammospheres significantly increased in MUC1‐positive cells and decreased in MUC1‐null cells in both cell lines (Figure 3E,F). Consistent with these results, the proportion of CD133‐positive (CD133⁺) cells increased in MUC1‐overexpressing cells and declined in MUC1‐null cells (Figure 3G,H). Moreover, extreme limiting dilution analysis (ELDA) was performed to examine clonal formation ability. The result revealed that stem cell frequency markedly increased with MUC1 overexpression and decreased upon MUC1 knockout in both NCI‐H69 (Figure 3I and Figure S2E,F (Supporting Information)) and NCI‐H526 cells (Figure 3J and Figure S2G (Supporting Information)). Notably, in vivo ELDA experiments in mice also demonstrated a reduced stem cell frequency in MUC1‐null cells (Figure 3K,L). Overall, these results validate that MUC1 induces expansion of CSLCs in SCLC. To confirm the relationship between stem cell properties and symmetric cell division, the immunofluorescence (IF) staining was performed in NCI‐H69 sphere‐forming cells, result showed that in symmetric division cells, the stem cell marker SOX2 was evenly spread in both daughter cells and localized with MUC1 (Figure S2H, Supporting Information). This result suggests that symmetric division promotes stem cell expansion.

### 2.4 Interaction of the Cytoplasmic Domain of MUC1 (MUC1–CD) with PP2A Depends on the CQC Motif

**Figure 4:**
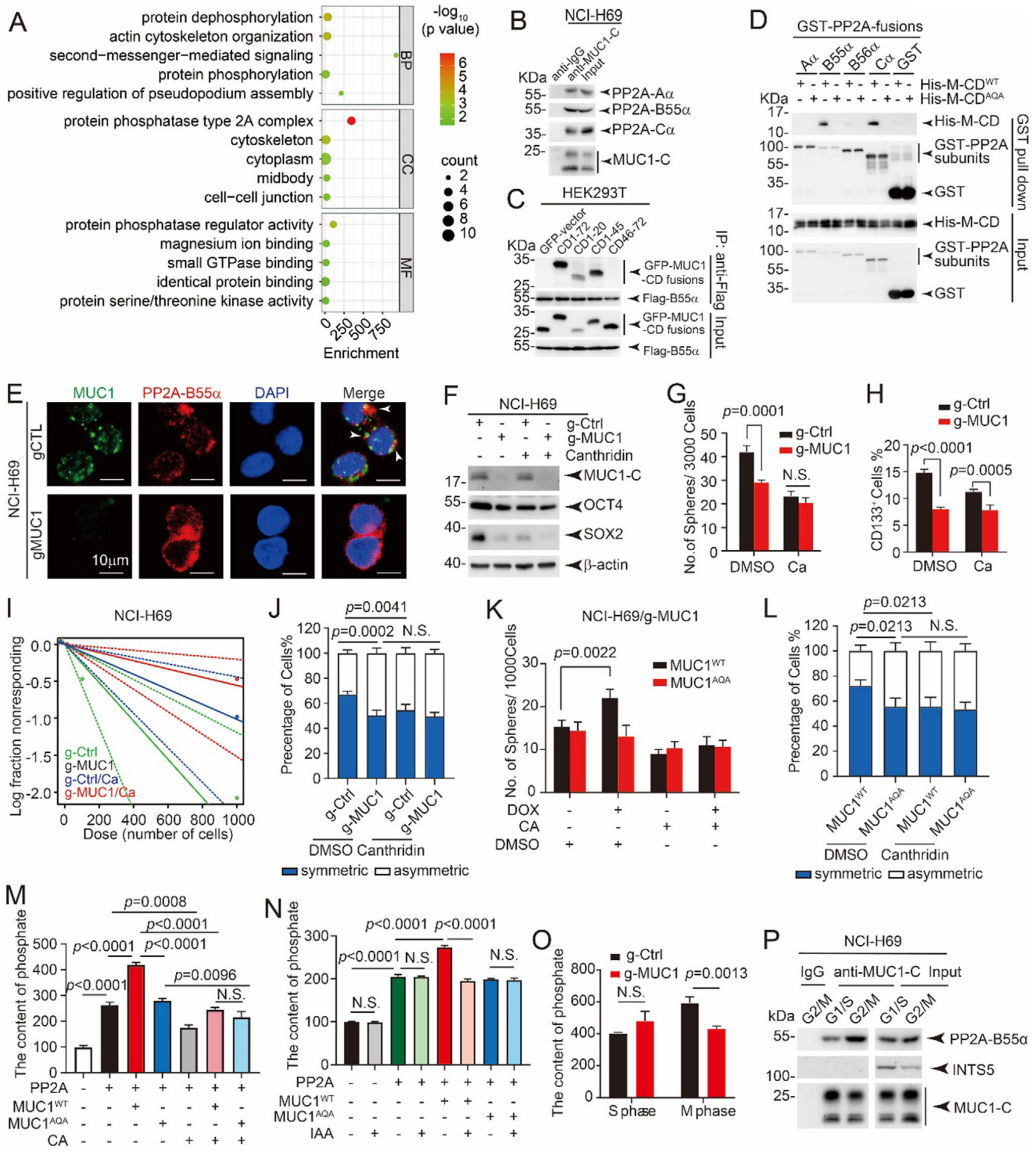
MUC1 and PP2A interaction enhances the activity of PP2A, thereby promoting symmetric division and tumor stemness. A) GO analysis of proteins in the G2‐M phase. B) co‐IP was performed in NCI‐H69 cells with anti‐MUC1‐C antibody, western blot was used to detect indicated proteins. C) HEK293T cells were transfected with Flag‐tagged PP2A‐B55α and different MUC1–CD truncates with GFP tags, the cells were collected after 48 h, and subjected to co‐IP with anti‐Flag antibody, western blot was performed to test GFP and Flag. D) In vitro GST pull‐down assays using purified proteins, and western blot was used to detect MUC1–CD and GST. E) Colocalization of MUC1‐C and PP2A‐B55α in cells was detected by IF. Scale bars: 10 µm. F) NCI‐H69/g‐Ctrl and NCI‐H69/g‐MUC1 cells were treated with dimethyl sulfoxide (DMSO) and 2 µm Cantharidin, respectively, for 48 h. Cells were collected, and western blot was performed to detect the protein levels of OCT4 and SOX2 stem cell factors. G) NCI‐H69/g‐Ctrl and NCI‐H69/g‐MUC1 cells were treated with DMSO and 2 µm CA, respectively, and subjected to sphere‐forming assay; the number of spheres was statistically analyzed. The data are presented as the mean ± SD from three independent experiments. H) NCI‐H69/g‐Ctrl and NCI‐H69/g‐MUC1 cells were treated with DMSO and 2 µm CA for 48 h, respectively, labeled with CD133–PE, and the proportion of CD133^+^ cells were detected by FCM assay. The data are presented as the mean ± SD from three independent experiments. I) ELDA was used to quantify CSLC frequencies in NCI‐H69/g‐Ctrl and g‐MUC1 cells after CA treatment. J) CSLCs were enriched by sphere‐forming assay using NCI‐H69/g‐Ctrl and NCI‐H69/g‐MUC1 cells, and then digested into individual cells and continued to be cultured with sphere‐forming medium, and then cells were treated with DMSO and 2 µm Cantharidin, respectively. After treated with 25 µm Blebbistatin for 48 h, cells were collected to perform IF assay to observe the distribution of NUMB and MUC1 in the cells. Statistically analyze the proportion of symmetric and asymmetric division patterns in the cells with different treatments. The data are presented as the mean ± SD from three independent experiments. K) Expression of MUC1^WT^ and MUC1^AQA^ mutants were induced by DOX in NCI‐H69/g‐MUC1 cells. Sphere‐forming assay was performed. Cells were treated with DMSO and 2 µm CA, respectively, and the number of spheres was statistically analyzed. The data are presented as the mean ± SD from three independent experiments. L) Expression of MUC1^WT^ and MUC1^AQA^ was induced in NCI‐H69/g‐MUC1 cells, followed by sphere‐forming assay to enrich cancer stem‐like cells, and the spheres were separated into individual cells, which continued to be cultured with sphere‐forming medium, and the cells were treated with DMSO and 2 µm Cantharidin, respectively, 25 µm Blebbistatin was added to all the cells, and after 48 h, IF assay was subjected to observe the distribution of NUMB and MUC1 in the cells and statistically analyze the proportion of symmetric and asymmetric division patterns in the cells with different treatments. The data are presented as the mean ± SD from three independent experiments. M) The PP2A heterotrimeric complex and MUC1–CD^WT^ and MUC1–CD^AQA^ were purified in vitro. PP2A was incubated with either MUC1–CD^WT^ or MUC1–CD^AQA^ in the presence of DMSO or CA, respectively, and phosphatase activity was assessed using a phosphatase activity assay kit. The data are presented as mean ± SD from three independent experiments. N) The PP2A heterotrimeric complex and MUC1–CD^WT^ and MUC1–CD^AQA^ were purified in vitro. PP2A was incubated with either MUC1–CD^WT^ or MUC1–CD^AQA^ in the presence of DMSO or IAA, respectively, and phosphatase activity was assessed using a phosphatase activity assay kit. The data are presented as mean ± SD from three independent experiments. O) NCI‐H69/g‐Ctrl and NCI‐H69/g‐MUC1 cells were sorted into G1/S and G2/M phase populations by flow cytometry. PP2A‐Cα was isolated using immunoprecipitation, and its phosphatase activity was measured using a phosphatase activity assay kit. The data are presented as mean ± SD from three independent experiments. P) Co‐IP was performed with antibody to MUC1‐C in NCI‐H69 cells at different phases of the cell cycle. The pellet was used to detect indicated proteins by western blot.

To investigate the mechanism by which MUC1 regulates cell division patterns, we overexpressed HA‐tagged MUC1 in HEK293T cells and analyzed interacting proteins using co‐immunoprecipitation (co‐IP) and mass spectrometry. Among a total of 526 MUC1‐interacting proteins, 34 were associated with cell cycle regulation (Figure S3A, Supporting Information). Gene ontology (GO) analysis of these proteins revealed significant enrichment in dephosphorylation‐related proteins, particularly PP2A (**Figures** **4**A and S3B (Supporting Information)). PP2A protein is a ternary complex consisting of an A subunit that serves as a backbone, a B subunit that recognizes substrates, and a C subunit that has a catalytic role. Co‐IP analysis in MUC1‐HA‐overexpressing HEK293T cells revealed that MUC1 interacts with Aα, B55α, B56α, and Cα of PP2A (Figure S3C, Supporting Information). To further validate the interaction between PP2A and MUC1, MUC1 and Flag‐tagged PP2A‐B55α subunits were coexpressed in HEK293T cells. Immunoprecipitation showed that Flag–PP2A‐B55α pulled down MUC1 along with the Aα and Cα subunits of PP2A (Figure S3D, Supporting Information). This interaction was further confirmed in both NCI‐H69 (Figure 4B) and HeLa 229 cells (Figure S3E, Supporting Information). To explore the binding site of MUC1 for PP2A, co‐IP was further performed in HEK293T cells coexpressing Flag‐tagged B55α and various GFP‐tagged truncations of MUC1–CD. Only the fragments containing amino acids 1–20 or 1–45, but not 46–72, were able to interact with B55α (Figure 4C), suggesting that the N‐terminal 1–20 region is critical for this interaction. We next performed in vitro GST pull‐down assays using GST‐tagged PP2A subunits and His‐tagged MUC1–CD. The data revealed that wild‐type MUC1–CD, but not the AQA mutant, bound to both the B55α and Cα subunits of PP2A (Figure 4D). These results indicate that the CQC motif is essential for the direct interaction between MUC1–CD and PP2A. Finally, immunofluorescence analysis demonstrated colocalization of PP2A‐B55α and MUC1–CD in NCI‐H69 cells, further supporting the interaction between the two proteins (Figure 4E).

### 2.5 Interface of MUC1 and PP2A Is Required for Elevation of PP2A Activity and Symmetric Cell Division

To validate whether PP2A affects the stemness of CSLCs in SCLC, the cells were treated with Cantharidin (CA), a PP2A inhibitor. CA treatment significantly reduced the protein levels of stemness‐associated factors (Figure 4F) and decreased mammosphere formation, particularly in MUC1‐positive cells (Figure 4G and Figure S4A (Supporting Information)). Flow cytometry analysis discovered a marked reduction in the proportion of CD133^+^ cells following CA treatment (Figure 4H and Figure S4B (Supporting Information)). Consistently, ELDA experiments demonstrated a substantial reduction in the frequency of CSLCs upon CA treatment (Figure 4I and Figure S4C (Supporting Information)). We next examined whether PP2A activity affects cell division patterns. Results showed that CA significantly reduced the proportion of symmetric divisions in MUC1‐positive cells (Figure 4J and Figure S4D (Supporting Information)). To further explore the importance of MUC1–PP2A interaction, MUC1^WT^ or MUC1^AQA^ were ectopically expressed, respectively, in NCI‐H69/g‐MUC1 cells. Sphere‐forming assay indicated that MUC1^WT^, but not MUC1^AQA^, significantly enhanced sphere‐forming capacity. This effect was suppressed by CA (Figure 4K and Figure S4E (Supporting Information)). Moreover, MUC1‐induced symmetric cell division was markedly suppressed by either CA or the AQA mutant (Figure 4L and Figure S4F (Supporting Information)). To determine whether MUC1 modulates the PP2A activity, we measured the phosphatase activity of PP2A following in vitro coincubating with either wild‐type MUC1–CD or the AQA mutant. Notably, wild‐type MUC1–CD, but not the AQA mutant, significantly increased the phosphatase activity of PP2A (Figure 4M,N). Furthermore, either CA (Figure 4M) or iodoacetamide, which blocks cysteine residues in MUC1–CD (Figure 4N), abrogated the stimulatory effect of MUC1–CD on PP2A activity. Interestingly, MUC1 significantly enhanced PP2A activity during the M phase, but not the S phase, of the cell cycle (Figure 4O). Constantly, co‐IP analysis revealed that the interaction between MUC1 and PP2A was stronger during the G2/M phase than in the G1/S phase (Figure 4P). These results confirm that the interface between MUC1 and PP2A is essential for elevation of PP2A activity and symmetric cell division.

### 2.6 PP2A Reduces NUMB Phosphorylation in MUC1‐Positive SCLC

**Figure 5:**
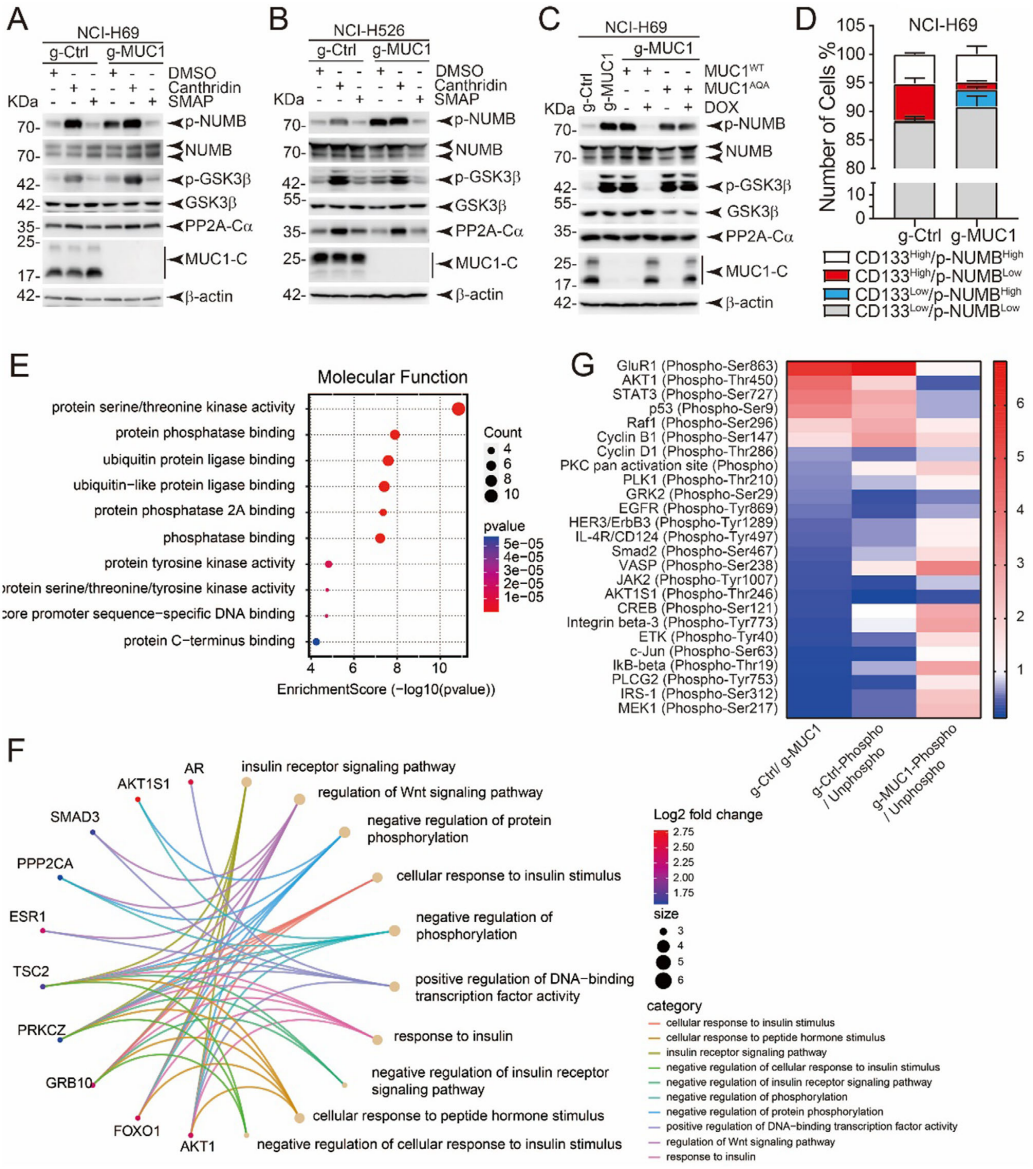
PP2A inhibits the phosphorylation of NUMB. A) NCI‐H69/g‐Ctrl and NCI‐H69/g‐MUC1 cells were treated with DMSO, 2 µm Cantharidin, and 5 µm SMAP for 48 h, respectively. Cells were collected, and subjected to western blot to detect the indicated proteins with β‐actin as an internal reference. B) NCI‐H526/g‐Ctrl and NCI‐H526/g‐MUC1 cells were treated with DMSO, 2 µm Cantharidin, and 5 µm SMAP for 48 h, respectively. Cells were collected to detect the indicated proteins levels by western blot with β‐actin as an internal reference. C) Expression of MUC1^WT^ and MUC1^AQA^ was induced in NCI‐H69/g‐MUC1 cells with DOX, and the cells were collected. Western blot was performed to detect the protein levels of indicated proteins, using β‐actin as an internal reference. D) NCI‐H69/g‐Ctrl and NCI‐H69/g‐MUC1 cells were labeled with both CD133 and p‐NUMB, and the proportion of CD133 and p‐NUMB positivity was detected by FCM assay and statistically analyzed. The data are presented as the mean ± SD from three independent experiments. E) Using NCI‐H69/g‐Ctrl and NCI‐H69/g‐MUC1 cells, the protein phosphorylation levels in the cells were compared, and the functions of proteins with higher phosphorylation levels after knockdown of MUC1 were analyzed. F) Cellular functions regulated by PP2A and PKCζ together. G) Comparison of phosphorylation levels of some proteins in NCI‐H69/g‐Ctrl and NCI‐H69/g‐MUC1 cells.

Given that NUMB determines cell fate,^[^ ^41^, ^42^, ^43^ ^]^ we first examined its protein levels and phosphorylation status. Data presented that MUC1 deficiency pointedly increased NUMB phosphorylation but had little, if any, effect on NUMB protein levels in both NCI‐H69 cells (**Figure** **5**A) and NCI‐H526 cells (Figure 5B). To confirm the phenomenon of MUC1‐induced PP2A activation, cells were treated with PP2A activator—SMAP. The phosphorylation levels of GSK3β and c‐Raf (two PP2A substrates) were dramatically reduced by MUC1, which was similar to SMAP treatment (Figures 5A,B and Figure S5A (Supporting Information)). These data suggest that MUC1 and SMAP are functionally analogous in enhancing PP2A activity. We further rescued MUC1^WT^ or MUC1^AQA^ into MUC1‐null NCI‐H69 cells and found the phosphorylation level of NUMB was decreased by overexpression of MUC1^WT^ rather than MUC1^AQA^ (Figure 5C). These data indicate that MUC1/PP2A mediates NUMB dephosphorylation. In support of this finding, NOTCH2 activity was augmented by MUC1 and SMAP, but reduced by MUC1 deficiency or CA treatment (Figure S5B, Supporting Information). To explore the NUMB phosphorylation in CSLCs, CD133^+^ cells were analyzed by flow cytometry and results showed that the proportion of CD133^High^/p‐NUMB^Low^ cells was significantly higher in g‐Ctrl cells, demonstrating the phosphorylation level of NUMB in CSLCs was repressed by MUC1 (Figure 5D and Figure S5C (Supporting Information)).

We hypothesized that PP2A binds and dephosphorylates NUMB. To our surprise, we found little if any interaction between PP2A and NUMB (data not shown). This result indicates that PP2A may affect other proteins that directly regulate NUMB phosphorylation. To identify potential mediators, we performed phosphorylation microarray analysis in NCI‐H69 cells. Consistent with our findings, the results showed that MUC1 enhanced PP2A activity, and many genes with reduced phosphorylation in MUC1‐positive cells were associated with PP2A‐binding proteins (Figure 5E). Notably, phosphorylation of the PKC family member PKCζ was significantly altered in response to MUC1 expression (Figure 5F,G).

### 2.7 PP2A Reduces PKCζ Phosphorylation in SCLC Cells

**Figure 6:**
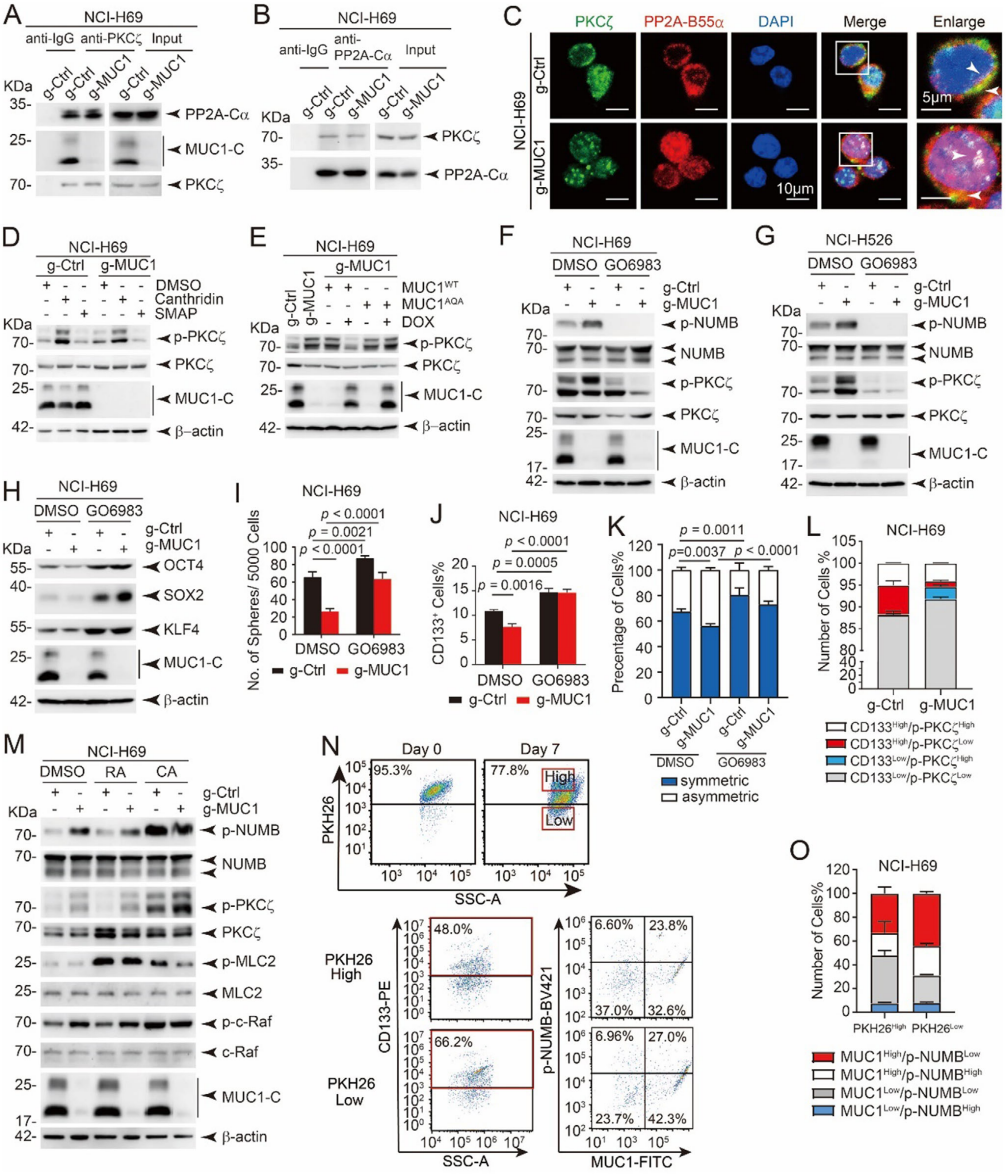
PP2A inhibits the phosphorylation of PKCζ, suppressing its activity and ultimately reducing the phosphorylation of NUMB, promoting symmetric division and tumor stemness. A) Co‐IP was performed in NCI‐H69/g‐Ctrl and NCI‐H69/g‐MUC1 cells with anti‐PP2A‐Cα antibody, western blot was used to detect indicated proteins. B) Co‐IP was performed in NCI‐H69/g‐Ctrl and NCI‐H69/g‐MUC1 cells with anti‐PKCζ antibody, western blot was used to detect indicated proteins. C) Colocalization of PKCζ and PP2A‐B55α in cells was detected by IF. Scale bars: 20 µm. D) NCI‐H69/g‐Ctrl and NCI‐H69/g‐MUC1 cells were treated with DMSO, 2 µm Canthridin, and 5 µm SMAP for 48 h, respectively. Cells were collected, and subjected to western blot to detect the indicated proteins with β‐actin as an internal reference. E) Expression of MUC1^WT^ and MUC1^AQA^ was induced by DOX in NCI‐H69/g‐MUC1 cells, respectively. Cells were collected to detect the indicated proteins by western blot with β‐actin as an internal reference. F) NCI‐H69/g‐Ctrl and NCI‐H69/g‐MUC1 cells were treated with DMSO, 5 µm GO6983 for 48 h, respectively. Cells were collected to detect the indicated proteins by western blot with β‐actin as an internal reference. G) NCI‐H526/g‐Ctrl and NCI‐H526/g‐MUC1 cells were treated with DMSO, 5 µm GO6983 for 48 h, respectively. Cells were collected to detect the indicated proteins by western blot with β‐actin as an internal reference. H) NCI‐H69/g‐Ctrl and NCI‐H69/g‐MUC1 cells were treated with DMSO and 5 µm GO6983 for 48 h, respectively. Cells were collected to detect the indicated proteins by western blot with β‐actin as an internal reference. I) NCI‐H69/g‐Ctrl and NCI‐H69/g‐MUC1 cells were treated with DMSO and 5 µm GO6983, respectively, and subjected to sphere‐forming assay; the number of spheres was statistically analyzed. The data are presented as the mean ± SD from three independent experiments. J) NCI‐H69/g‐Ctrl and NCI‐H69/g‐MUC1 cells were treated with DMSO and 5 µm GO6983 for 48 h, respectively, labeled with CD133‐PE, and the proportion of CD133^+^ cells was detected by FCM assay. The data are presented as the mean ± SD from three independent experiments. K) CSLCs were enriched by sphere‐forming assay using NCI‐H69/g‐Ctrl and NCI‐H69/g‐MUC1 cells, and then digested into individual cells and continued to be cultured with sphere‐forming medium; and then cells were treated with DMSO and 5 µm GO6983, respectively. After treated with 25 µm Blebbistatin for 48 h, cells were collected to perform IF assay to observe the distribution of NUMB and MUC1 in the cells. Statistically analyze the proportions of symmetric and asymmetric division modes in the cells with different treatments. The data are presented as the mean ± SD from three independent experiments. L) NCI‐H69/g‐Ctrl and NCI‐H69/g‐MUC1 cells were labeled with both CD133 and p‐PKCζ, and the proportion of CD133 and p‐PKCζ positivity was detected by FCM assay and statistically analyzed. The data are presented as the mean ± SD from three independent experiments M) NCI‐H69/g‐Ctrl and NCI‐H69/g‐MUC1 cells were treated with DMSO, 2 µm Ralphin acete, and 2 µm Canthridin for 48 h, respectively. Cells were collected, and subjected to western blot to detect the indicated proteins with β‐actin as an internal reference. N,O) 7 days after PKH26 labeled, NCI‐H69 cells were flow‐sorted to obtain two fractions of cells with high PKH26 and low PKH26, and then labeled with CD133, MUC1, p‐NUMB, and p‐PKCζ at the same time, which were detected by FCM assay and statistically analyzed. The data are presented as the mean ± SD from three independent experiments.

To identify the specific PKC protein responsible for regulating NUMB phosphorylation, cells were treated with inhibitors targeting different members of the PKC family. Notably, inhibition of PKCζ by GO6983 substantially reduced NUMB phosphorylation (Figure S5D, Supporting Information). To explore the relationship between PP2A and PKCζ, co‐IP was performed in NCI‐H69 cells and revealed that PP2A‐Cα interacts with PKCζ in a MUC1‐independent manner (**Figures** **6**A,B and S5E (Supporting Information)). This result is supported by immunofluorescence (Figure 6C) and PLA data (Figure S5F, Supporting Information), which demonstrated colocalization of PP2A and PKCζ within cells. Constantly, knockdown of PP2A‐B55α not only induced the phosphorylation of both NUMB and PKCζ(Figure S5G, Supporting Information), but also reduced sphere formation and the proportion of CD133^+^ cells (Figure S5H,I, Supporting Information). Moreover, the phosphorylation level of PKCζ was enhanced by PP2A inhibitor but reduced by PP2A activator (Figure 6D). Similarly, the phosphorylation of PKCζ was prominently declined by overexpression of MUC1^WT^ but not MUC1^AQA^ (Figure 6E). To validate the role of PKCζ in regulating NUMB, cells were treated with the PKCζ inhibitor GO6983, which significantly suppressed NUMB phosphorylation in both NCI‐H69 and NCI‐H526 cells (Figure 6F,G). To determine whether the MUC1–PP2A axis functions across other SCLC subtypes, we investigated phosphorylation levels of PKCζ and NUMB in cell lines representing distinct subtypes–NCI‐H69 (SCLC‐A), NCI‐H526 (SCLC‐P) and NCI‐H82 (SCLC‐N). MUC1–PP2A–PKCζ–NUMB signaling axis was significantly activated in MUC1‐overexpressed NCI‐H69 and NCI‐H526 cell lines (Figures 5A,B and 6F,G). This phenomenon was confirmed in NCI‐H82 cell line when enforced expression of MUC1 (Figure S6A, Supporting Information). These results demonstrate that the MUC1–PP2A axis is a conserved regulatory mechanism across SCLC subtypes. To determine whether this effect also occurs in other cancer types, we detected the phosphorylation of NUMB and PKCζ in cell lines of breast cancer and cervical cancer which overexpress MUC1. We observed consistent results that MUC1 activated PP2A and suppressed phosphorylation of NUMB and PKCζ (Figure S6B, Supporting Information). These findings suggest that the MUC1–PP2A signaling axis may be a conserved mechanism in various cancers.

### 2.8 Inhibition of PKCζ Promotes Symmetric Division and Expands CSLCs

The role of PKCζ in regulating stemness was further supported by evidence that treatment with the PKCζ inhibitor GO6983 significantly increased the levels of stem‐cell‐associated proteins (Figure 6H), enhanced mammosphere formation (Figure 6I and Figure S6C (Supporting Information)), and elevated the proportion of CD133⁺ cells (Figure 6J and Figure S6D (Supporting Information)), suggesting that inhibition of PKCζ promotes the enrichment of stem‐like characteristics. In support of this, the proportion of symmetric cell divisions was significantly increased upon PKCζ inhibition (Figure 6K and Figure S6E (Supporting Information)). In line with reduced NUMB phosphorylation, the percentage of CD133^High^/pPKCζ^Low^ in CSLCs was significantly elevated in g‐Ctrl cells (Figure 6L and Figure S6F (Supporting Information)). To explore whether other phosphatases might also contribute to MUC1‐related phosphorylation of NUMB and PKCζ, cells were treated with Raphin1 acetate (RA), a specific inhibitor for protein phosphatase 1 (PP1). Data showed that MUC1 mediated reduction of phosphorylation of PKCζ and NUMB was blocked by PP2A inhibitor (CA) rather than PP1 inhibitor (RA), indicating that MUC1‐mediated regulation of NUMB and PKCζ phosphorylation is dependent on PP2A, but not PP1 (Figure 6M). To further characterize proliferative behavior, spheroid‐forming cells were separated into fast‐proliferating (PKH26^Low^) and slow‐proliferating (PKH26^High^) populations based on PKH26 dye intensity (Figure 6N top). Interestingly, compared with PKH26^High^ cells, the PKH26^Low^ cells contained a higher proportion of CD133^+^ cells and exhibited increased MUC1^High^/pNUMB^Low^ (Figure 6N,O bottom and Figure S6G (Supporting Information)). These findings suggest that CSLCs exhibiting a MUC1^High^/pNUMB^Low^ phenotype preferentially undergo symmetric division and rapid expansion, contributing to increased CD133⁺ cell populations. Overall, these results demonstrate that MUC1 interacts with PP2A to enhance its activity, thereby reducing PKCζ and NUMB phosphorylation, promoting symmetric division, and expanding the CSLC compartment.

### 2.9 Combined Etoposide Treatment and Targeted Inhibition of the MUC1–PP2A Pathway Suppress Tumor Growth in SCLC

**Figure 7:**
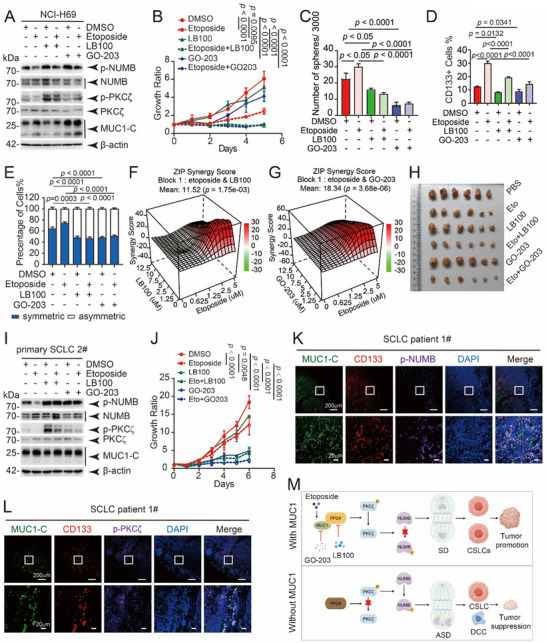
Targeted inhibition of MUC1–PP2A combined with etoposide inhibits tumor growth. A) NCI‐H69 cells were treated with DMSO, 5 µm etoposide, 10 µm LB100, etoposide + LB100, 5 µm GO‐203, and etoposide + GO‐203 for 48 h, respectively. Cells were collected to detect the indicated proteins by western blot with β‐actin as an internal reference. B) NCI‐H69 cells treated with DMSO, 5 µm etoposide, 10 µm LB100, etoposide + LB100, 5 µm GO‐203, and etoposide + GO‐203 were subjected to cell counting kit 8 (CCK‐8) assays to assess cell viability. The data are presented as the mean ± SD from three independent experiments. C) NCI‐H69 cells were treated with DMSO, 5 µm etoposide, 10 µm LB100, etoposide + LB100, 5 µm GO‐203, and etoposide + GO‐203, respectively, and subjected to sphere‐forming assay; the number of spheres was statistically analyzed. The data are presented as the mean ± SD from three independent experiments. D) NCI‐H69 cells were treated with DMSO, 5 µm etoposide, 10 µm LB100, etoposide + LB100, 5 µm GO‐203, and etoposide + GO‐203 for 48 h, respectively, labeled with CD133–PE. The proportion of CD133^+^ cells was detected by FCM assay and analyzed statistically. The data are presented as the mean ± SD from three independent experiments. E) CSLCs were enriched by sphere‐forming assay using NCI‐H69 cells, and then digested into individual cells and continued to be cultured with sphere‐forming medium, and then cells were treated with DMSO, 5 µm etoposide, 10 µm LB100, etoposide + LB100, 5 µm GO‐203, and etoposide + GO‐203, respectively. After treated with 25 µm Blebbistatin for 48 h, cells were collected to perform IF assay to observe the distribution of NUMB and MUC1 in the cells. Statistically analyze the proportion of symmetric and asymmetric division patterns in the cells with different treatments. The data are presented as the mean ± SD from three independent experiments. F,G) Drug synergy experiments of etoposide with LB100 (F) and etoposide with GO‐203 (G), synergy score was calculated via the ZIP model. H) NCI‐H69 cells were subjected to mouse xenograft assays. Mice were treated with DMSO, etoposide (20 mg kg^−1^, once every three days), LB100 (2.5 mg kg^−1^, every day), combination of etoposide (20 mg kg^−1^, once every three days) and LB100 (2.5 mg kg^−1^, every day), GO‐203 (15 mg kg^−1^, every day), or combination of etoposide (20 mg kg^−1^, once every three days) and GO‐203 (15 mg kg^−1^, every day) for 15 days. I) Patient primary cells were treated with DMSO, 0.1 µm etoposide, 2.5 µm LB100, etoposide + LB100, 3 µm GO‐203, and etoposide + GO‐203 for 48 h, respectively. Cells were collected to detect the indicated proteins by western blot with β‐actin as an internal reference. J) Patient primary cells treated with DMSO, 0.1 µm etoposide, 2.5 µm LB100, etoposide + LB100, 3 µm GO‐203, and etoposide + GO‐203 subjected to CCK‐8 assays to assess cell viability. The data are presented as the mean ± SD from three independent experiments. K,L) Colocalization of MUC1‐C and p‐NUMB (K), MUC1‐C and CD133, MUC1‐C and p‐PKCζ (L) in tissues derived from dissected tumors of SCLC patients were detected by IF. Scale bars: 200 µm. M) Schematic overview of the proposed working model. In the G2/M phase, MUC1 interacts with and activates PP2A, leading to reduced phosphorylation of PKCζ and, subsequently, decreased phosphorylation of NUMB. Reduced NUMB phosphorylation promotes symmetric division of CSLCs, thereby expanding the CSLC population and contributing to tumor progression. Targeted inhibition of the MUC1–PP2A axis using LB100 or GO‐203, in combination with etoposide, enhances PKCζ activity and restores NUMB phosphorylation, ultimately suppressing CSLC expansion and tumor growth both in vitro and in vivo.

Our study reveals that the MUC1–PP2A–pPKCζ–pNUMB signaling pathway promotes symmetric division and stemness in SCLC cells. To assess the clinical relevance of this pathway, the cells were treated with etoposide and combined with LB100 targeting to PP2A or GO‐203 targeting to MUC1, respectively. The result suggested that phosphorylation of NUMB and PKCζ was reduced by etoposide and restored upon cotreatment with either LB100 or GO‐203 (**Figure** **7**A). Cell proliferation assays revealed that the combination of etoposide with either inhibitor significantly suppressed cell growth compared to monotherapies (Figure 7B), indicating a synergistic effect. Consistently, mammosphere formation was enhanced by etoposide alone but significantly reduced by cotreatment with LB100 or GO‐203 (Figure 7C and Figure S7A (Supporting Information)). A similar result was found in the frequency of CD133^+^ cells (Figure 7D and Figure S7B (Supporting Information)). These results suggest that etoposide‐induced CSLC enrichment can be reversed by targeted inhibition of MUC1 or PP2A. Supporting these findings, etoposide increased the proportion of symmetric divisions, which was significantly reduced by cotreatment (Figure 7E and Figure S7C (Supporting Information)).

To validate the combined effect of etoposide with LB100 or GO‐203, drug synergy assay was performed (Figure 7F,G). The results demonstrated that either combination of etoposide and LB100 or combination of etoposide–GO‐203 exhibited synergy scores exceeding 10 (calculated via the ZIP model), indicating synergistic effects of combination treatment with etoposide and MUC1–PP2A targeted therapy. We further evaluated the in vivo efficacy of this combination therapy using NCI‐H69 xenografts in nude mice. Although etoposide alone moderately suppressed tumor growth, its combination with either LB100 or GO‐203 produced significantly greater tumor inhibition (Figure S7D–H, Supporting Information) and effectively prevented tumor regrowth observed in the etoposide‐alone group (Figure S7D, Supporting Information). Therapeutic efficacy was further validated in primary SCLC‐patient‐derived cells. Again, phosphorylation levels of NUMB and PKCζ were decreased with etoposide treatment and rescued by combination with etoposide and LB100 or GO‐203 (Figure 7I). Cell growth was also sufficiently repressed by combination treatment of etoposide and LB100 or GO‐203 (Figure 7J). Observing tumor sections from patients, we found that MUC1 suggestively colocalized with CD133 rather than p‐NUMB and p‐PKCζ (Figure 7K,L and Figure S8A–D (Supporting Information)). These findings suggest that high MUC1 expression in patient tumors may suppress phosphorylation of NUMB and PKCζ, thereby enriching CSLCs. Thus, targeting the MUC1–PP2A axis represents a promising therapeutic strategy for eliminating CSLCs in SCLC.

## 3 Discussion

CSC expansion drives key oncogenic processes including tumor initiation, metastatic dissemination, and chemoresistance. Previous researches have demonstrated that CSCs depend heavily on MUC1 for self‐renewal,^[^ ^31^, ^33^, ^34^, ^44^ ^]^ highlighting a critical role for MUC1 in CSC expansion. However, its role in regulating CSC division patterns has remained unclear. Here, we demonstrate that MUC1 is highly expressed during the G2/M phase, where it promotes symmetric division and expansion of CSLCs in SCLC. And our finding suggests that symmetric division promotes stem cell expansion. The 526 proteins interacting with MUC1 obtained by co‐IP and mass spectrometry experiments were categorized by KEGG analysis. We find there 34 proteins related to cell cycle (Figure S3A, Supporting Information). We then performed GO analysis on the 34 cell‐cycle‐related proteins and found that PP2A was the most significantly enriched. Mechanistic studies reveal that MUC1 interacts with PP2A and enhancing its activity. This leads to reduced phosphorylation of PKCζ, ultimately decreasing the phosphorylation of NUMB. The reduction of NUMB phosphorylation promotes symmetric division in CSLCs, thereby enriching their population and contributing to tumorigenesis. Both pharmacological and genetic strategies validate that targeted‐blocking the MUC1–PP2A axis significantly elevates PKCζ activity and phosphorylation of NUMB, ultimately suppressing CSLCs and tumor growth in vitro and in vivo. These findings uncover a novel mechanism by which MUC1 drives CSLC expansion in SCLC through the modulation of PP2A activity and NUMB phosphorylation (Figure 7M).

In agreement with our discovery, MUC1 has been implicated in the regulation of CSCs. MUC1‐C promotes lineage plasticity, driving progression to neuroendocrine prostate cancer by suppressing the p53 pathway and inducing the Yamanaka pluripotency factors (octamer binding transcription factor 4 (OCT4), SOX2, Krüppel‐like factor 4 (KLF4), and MYC).^[^ ^45^ ^]^ Our previous research also demonstrates that MUC1 enhances breast cancer stemness by reducing the TCA cycle and inducing human epidermal growth factor receptor 2 (HER2)/EGFR dimerization, leading to lineage plasticity in HER2‐positive mammary tumors.^[^ ^27^ ^]^ In the present study, we revealed that MUC1 protein levels are elevated during the G2/M phase in SCLC cells and promote CSLC expansion by shifting cell division toward symmetric modes. Noteworthy, we also found that CD133^High^/MUC1^High^ cells could differentiate into CD133^High^/MUC1^Low^ cells, whereas the CD133^High^/MUC1^Low^ cells only produced CD133^Low^/MUC1^Low^ cells, suggesting the role of MUC1in maintenance of CSLC cells. The regulation of stem cell division patterns is crucial for embryogenesis, development, and tissue regeneration.^[^ ^18^, ^46^ ^]^ During wound healing and regeneration, adult stem cells can also be converted to symmetric division, restoring stem cells depleted by injury repair.^[^ ^46^, ^47^, ^48^ ^]^ Consistent with our findings, others have reported that MUC1 contributes to self‐renewal and tissue regeneration.^[^ ^49^ ^]^ These findings suggest that MUC1 may also facilitate injury repair by promoting symmetric stem cell division.

PP2A‐B55, a member of the protein serine/threonine phosphatase family, targets a broad range of cellular substrates for dephosphorylation and plays essential roles in cell cycle regulation. During the G2 phase, PP2A‐B55 holoenzymes inhibit the G2‐to‐M transition by suppressing maturation‐promoting factors formed by cyclin‐dependent kinase 1 (CDK1) and cyclin B;^[^ ^50^, ^51^ ^]^ in the early M phase, PP2A‐B55 activity is inhibited by the Greatwall kinase; once chromosomes are correctly attached to the fibers of the spindle, the anaphase‐promoting complex is activated which inhibits Greatwall and thereby reactivates PP2A‐B55 to ensure proper mitotic exit.^[^ ^52^ ^]^ In this study, we found MUC1 has little, if any effect on the protein level of PP2A‐Cα and PP2A‐B55α (Figure 5C and Figure S5G, Supporting Information), but M‐phase‐localized MUC1 interacts with the subunit of PP2A‐B55α, enhancing PP2A activity. This interaction appears to regulate the division pattern of SCLC stem‐like cells. The role of PP2A in cancer is context‐dependent and remains controversial, previous studies have suggested that PP2A is a cancer‐suppressor protein.^[^ ^53^, ^54^ ^]^ Nevertheless, other research indicates that PP2A can promote tumor growth through various mechanisms. For example, PP2A dephosphorylates macrophage stimulatory protein (MST1/2) and activates Yes‐Associated Protein (YAP), leading to tumor growth;^[^ ^55^ ^]^ it has also been found that PP2A with the B55α subunit stabilizes MYC, promoting tumor growth.^[^ ^56^ ^]^ Our findings reveal that PP2A dephosphorylates PKCζ, contributing to CSLC expansion in MUC1‐positive SCLC. This finding provides further support for PP2A's role in promoting tumor growth.

NUMB is a membrane‐associated protein that promotes the degradation of NOTCH intracellular domain, thereby preventing the transcription of NOTCH target genes.^[^ ^57^, ^58^, ^59^ ^]^ It is also believed to play a role in regulating the stemness of tumor stem cells.^[^ ^59^, ^60^ ^]^ As a cell fate determinant, the knockdown of NUMB disrupts cell cycle progression, impairs neural differentiation, and causes excessive proliferation of progenitor cells.^[^ ^61^, ^62^ ^]^ Studies in chronic granulocytic leukemia have shown that the reduction of NUMB protein levels results in an increase in stem cell populations.^[^ ^63^ ^]^ During cell division, the phosphorylation of NUMB influences its localization, leading to asymmetric cell division.^[^ ^64^, ^65^ ^]^ Given that NUMB is not only associated with the NOTCH signaling pathway that affects cell stemness, but also involved in regulating the activity of Par complex that affects the formation of cell polarity, it was commonly used as a marker of cell symmetric division by multiple lines of study.^[^ ^19^, ^66^ ^]^ Furthermore, phosphorylation at the S276 site affects the interaction of NUMB with β‐integrins, p120 catenin, and α‐adaptin, thereby attenuating E‐cadherin endocytosis to promote apicobasal polarity.^[^ ^67^, ^68^ ^]^ We found that MUC1 modulates NUMB phosphorylation rather than alters NUMB protein levels. Our data indicate that PP2A reduces NUMB phosphorylation at the S276 site, potentially affecting its symmetric localization during cytokinesis. Surprisingly, co‐IP showed little, if any, interaction between PP2A and NUMB. Thus, we guess PP2A might affect other protein that directly regulates NUMB phosphorylation. By phosphorylation microarray, we found that PKCζ was involved in the regulation of phosphorylation of NUMB. This is further confirmed by using PKCζ inhibitor. The mechanism of how PP2A selective dephosphorylated of PKCζ may be due to the structural differences between PKCζ and other PKC family members, or PKCζ but not other PKC family members are involved in the regulation of NUMB phosphorylation and cell polarity during cell division. Previous studies in *Drosophila* have shown that PKCζ‐mediated phosphorylation of NUMB affects the asymmetric fate of stem cells and PKCζ directly interacts with NUMB to modulate its phosphorylation.^[^ ^69^, ^70^, ^71^ ^]^ In line with this, we find that PKCζ phosphorylates NUMB and promotes asymmetric division of CSLCs. Inhibition of PKCζ activity results in decreased NUMB phosphorylation and increased number of CSLCs. Moreover, co‐IP assays revealed an interaction between PP2A and PKCζ, where PP2A‐mediated dephosphorylation downregulated PKCζ activity (Figure 6A–G). This is supported by our observation that both PP2A inhibition and B55α subunit knockdown increase PKCζ phosphorylation. Interestingly, MUC1, previously reported to stabilize MYC and promote NOTCH pathway activation, thereby promoting cancer.^[^ ^38^ ^]^ PKCζ phosphorylates MYC at the Ser373 site, promoting its degradation.^[^ ^72^ ^]^ We found that MUC1 constrains PKCζ activity by enhancing PP2A activation, which may also increase MYC stability. The dual role of PKCζ as both tumor promotor and tumor suppressor remains controversial across malignancies. In pancreatic cancer, PKCζ activates signal transducer and activator of transcription 3 (STAT3) to promote tumor cell growth and metastasis.^[^ ^73^ ^]^ Conversely, numerous studies have also indicated that PKCζ exerts an inhibitory effect on tumors. In a glucose‐deficient environment, PKCζ inhibits metabolic reprogramming in colorectal cancer cells and suppresses tumor cell growth.^[^ ^74^ ^]^ Additionally, PKCζ phosphorylates adenosine deaminase acting on RNA 2 (ADAR2), which inhibits the cellular secretion of miR‐200, thereby suppressing tumor metastasis.^[^ ^75^ ^]^ Our findings indicate that MUC1 enriches CSLCs by inhibiting PKCζ activity, supporting the tumor suppressor role of PKCζ.

The clinical treatment of SCLC primarily relies on chemotherapy using platinum‐based drugs and etoposide.^[^ ^76^ ^]^ However, patients with SCLC frequently develop drug resistance and experience rapid tumor recurrence.^[^ ^3^, ^4^ ^]^ Targeting CSLCs may effectively mitigate drug resistance and reduce posttreatment relapse in SCLC. Our data indicate that etoposide alone increases MUC1 protein levels and activates the MUC1–PP2A–PKCζ–NUMB pathway, resulting in an upregulation of the stem cell population (Figure 7A). Multiple lines of evidence indicate that LB100 and its derivatives inhibit tumor growth in various cancer models.^[^ ^77^, ^78^, ^79^ ^]^ Constantly, our study revealed that treatment with LB100 resulted in a reduction of MUC1‐C. Furthermore, combining etoposide with targeted inhibition of MUC1 (GO‐203) or PP2A (LB100) effectively reduced stemness in SCLC cells and significantly suppressed tumor growth. Importantly, analysis of SCLC patient tissues revealed a negative correlation between MUC1 expression and the phosphorylation levels of PKCζ and NUMB (Figure 7K,L).

SCLC is categorized into different subtypes based on the expression levels of the transcription factors achaete‐scute complex like protein 1 (ASCL1), neuronal differentiation 1 (NEUROD1), and POU domain, class 2, transcription factor 3 (POU2F3), as well as their inflammatory characteristics.^[^ ^80^ ^]^ Recognition of subtype‐specific molecular profiles can facilitate the development of novel targeting strategies. Although dominant molecular subtypes can be distinguished in most cases in SCLC, there is often significant heterogeneity in pathology and phenotypic switching may occur.^[^ ^81^ ^]^ It has been found that SCLC‐A subtype can be converted to SCLC‐N subtype and further to SCLC‐I subtype under MYC drive.^[^ ^82^, ^83^, ^84^ ^]^ Single‐cell sequencing analysis shows that MUC1 expression is the most prominent in the SCLC‐A subtype and normal epithelial stromal cells (Figure 1E–G). Analysis of SCLC transcriptome data revealed high expression of MUC1 and PP2A in the SCLC‐A, SCLC‐N, and SCLC‐P subtypes, accompanied with lower levels of NUMB and PKCζ (Figure 1G and Figure S8E (Supporting Information)), which is consistent with previous report.^[^ ^38^ ^]^ But the high expression of MUC1 was only considerably associated with poor prognosis in patients with SCLC‐A subtype. SCLC‐A and SCLC‐N subtypes are categorized as neuroendocrine variants. SCLC‐A is characterized by high expression of ASCL1 and SCLC‐N is characterized by high expression of NEUROD1, both of which are highly proliferative, invasive, and drug‐resistant. Both SCLC‐P and SCLC‐I types are associated with inflammatory signaling and are non‐neuroendocrine. MUC1‐C was found to upregulate the expression of ASCL1 and NEUROD1 through MYC activation.^[^ ^38^ ^]^ Meanwhile, MUC1 is involved in promoting inflammation.^[^ ^85^ ^]^ MUC1 activates PP2A and inhibits downstream protein phosphorylation in three different subtypes of SCLC cell lines, suggesting that the MUC1–PP2A axis is a conserved regulatory mechanism across all SCLC subtypes. According to the above results, we inferred that MUC1 is a distinguished biomarker for tumor growth and treatment of SCLC. Overall, our findings regarding the MUC1–PP2A pathways in SCLC cells illuminate a potential therapeutic strategy to target CSCs for the treatment of SCLC.

## 4 Experimental Section

### Bioinformatic Analysis

The small cell lung cancer genomics data sets were analyzed to discover genetic expression alterations of MUC1 within the GEO database. The single‐cell dataset for human small cell lung cancer was obtained from cellxgene (https://cellxgene.cziscience.com/collections/62e8f058‐9c37‐48bc‐9200‐e767f318a8ec), resulting in total 77 143 cells (filtered by disease “small cell lung carcinoma”). The trajectory analysis was performed by Monocle. *t*‐test was used to test the expression level difference between two groups.

### Cell Culture

HeLa 229, NCI‐H69, NCI‐H526, and NCI‐H82 were maintained in RPMI1640 supplemented with 10% fetal bovine serum (Gibco, Grand Island, NY, USA) in a 5% CO_2_ incubator at 37 °C. HEK293T cells were cultured in Dulbecco's modified Eagle medium (DMEM) containing 10% fetal bovine serum in a 5% CO_2_ incubator at 37 °C.

### Drugs and Antibodies

The following drugs and antibodies were used in the experiments: CA, IAA, SMAP, GO6983, LB100, etoposide (TargetMol, China), anti‐MUC1 antibody (Cell Signaling Technology, Danvers, MA, USA), anti‐Oct4 antibody (Cell Signaling Technology, Danvers, MA, USA), anti‐Sox2 antibody (Cell Signaling Technology, Danvers, MA, USA), anti‐Klf4 antibody (Cell Signaling Technology, Danvers, MA, USA), anti‐Nanog antibody (Cell Signaling Technology, Danvers, MA, USA), anti‐NUMB antibody (Proteintech Group, Chicago, IL, USA), anti‐PKCζ antibody (Cell Signaling Technology, Danvers, MA, USA), anti‐phospho‐NUMB antibody (Cell Signaling Technology, Danvers, MA, USA), anti‐phospho‐PKCζ antibody (Cell Signaling Technology, Danvers, MA, USA), anti‐PP2A‐Cα antibody (Merck Millipore, Billerica, MA, USA), anti‐PP2A‐Aα antibody (Cell Signaling Technology, Danvers, MA, USA), anti‐PP2A‐B55α antibody (Cell Signaling Technology, Danvers, MA, USA), anti‐PP2A‐B56α antibody (Cell Signaling Technology, Danvers, MA, USA), anti‐GFP antibody (Cell Signaling Technology, Danvers, MA, USA), anti‐Flag antibody (Cell Signaling Technology, Danvers, MA, USA), anti‐GST antibody (Cell Signaling Technology, Danvers, MA, USA), anti‐β‐actin antibody (Merck Millipore, Billerica, MA, USA), PE‐conjugated mouse anti‐human CD133/1 (AC133) (Mitenyi Biotec, Bergisch Gladbach, Germany).

### Plasmids and Transfection

MUC1‐deficient HeLa229, NCI‐H69, and NCI‐H526 cells were established using a CRISPR/Cas9 system. The MUC1 gRNA sequences included 5′‐GCTGCTCCTCACAGTGC‐3′ targeting the first exon. The viral vectors were produced in HEK293T cells as described. For the MUC1‐induced expression, the full length of MUC1 with HA tag at the C‐terminal was cloned into pInducer vector. For MUC1 overexpression, the full length of MUC1 with HA tag at the C‐terminal was cloned into pIRESpuro2 vector. Transient transfections were performed with Nano293T (NCM, C500T‐1, China) or Hilymax DNA Transfection Reagent (Dojindo, H357, Japan) according to the manufacturer's instructions.

### Extreme Limiting Dilution Assay In Vivo

Female 6–8 weeks old athymic nude mice (BALB/c‐nu/nu) were injected with NCI‐H69 cells ranging from 6.25 × 10^5^ to 1 × 10^7^ cells. After 14 days of culture, the mice that showed the size of the xenograft reached 4 mm in diameter were recorded as positive, or else were recorded as negative. Statistics were performed by the ELDA online tool (https://bioinf.wehi.edu.au/software/elda/).

### Western Blot

Cells were collected by trypsinization and resuspended in NETN150 lysis buffer (NaCl 150 mm, NP40 0.5%, Tris 20 mm pH 8.0, EDTA 1 mm) with protease inhibitor cocktails and phosphatase inhibitor Cocktail (TargetMol, China). The protein lysates were separated by sodium dodecyl sulfate–polyacrylamide gel electrophoresis (SDS‐PAGE) and transferred to the nitrocellulose membrane (Millipore, USA). After blocking in 5% nonfat milk, the membrane was incubated sequentially with primary antibodies overnight and HRP‐linked secondary antibodies. Chemiluminescence detection was with ECL (NCM, China).

### S Phase Synchronization of the Cell Cycle—Thymidine (TdR) Double Blockade

This method used excess TdR to block DNA synthesis, and in order to enhance the effect of cell synchronization, double TdR blockades were often used. HeLa229 cells were inoculated with 4 × 10^5^ cells in a 6 cm dish. After 24 h, 4 mm Thymidine was added, blocked for 12 h, the supernatant was aspirated, washed 3 times with precooled phosphate‐buffered saline (PBS) to make sure that there was no residue of Thymidine, fresh medium was added, and incubation was continued. 10 h later, Thymidine was added again, and blocked for 12 h, at which time more than 98% of the cells obtained were in S phase by flow assay. The cells were washed3 times with precooled PBS to ensure that there was no residue of Thymidine, and fresh medium was added to release the cells from S phase, and the cells were collected at different time points to obtain cells of different phases.

### M Phase Synchronization of the Cell Cycle

Cells were first blocked in S phase and then released and allowed to grow for 2 h, then 0.1 µg mL^−1^ of Nocodazol was added and incubated for 10 h. By mechanically shaking the Petri dish, or by gently blowing the medium with a pipette, the cells that floated up were M phase cells. 10 mL of PBS was added, the above steps were repeated, the medium was mixed with PBS, and centrifuged at 600 *g* for 5 min to obtain M phase cells. The cells were then washed twice with 10 mL of PBS, and the cells were reinoculated in the culture dish, different cells could be harvested at different time points. G1 phase cells could be obtained after 2 h.

### Sphere Formation Assay

Cells were plated in 24‐well ultralow attachment plates (Corning, USA) in DMEM/F12 (Basal Media, China) serum‐free medium containing 0.4% bovine serum albumin (BSA, Basal Medium, China), supplemented with 20 ng mL^−1^ epithelial growth factor (PerpoTech, Rocky Hill, NJ), 20 ng mL^−1^ basic fibroblast growth factor (PerpoTech, Rocky Hill, NJ), 50 µg mL^−1^ insulin (LABLEAD, China), and 1 × B27 (Gibco, USA) which was described previously. Cells were incubated for 4–6 days, and number of mammospheres (>50 µm in diameter) was counted under a microscope (Nikon Eclipse Ti, Tokyo, Japan).

### Flow Cytometry Assay

The expression of cell surface marker CD133 on cells was analyzed by flow cytometry assay. Briefly, cells were suspended in PBS containing 2% BSA. Combinations of CD133–PE or their respective isotype controls were added to the cell suspension at the concentrations recommended by the manufacturer and then incubated at 4 °C in the dark for 30 min. The labeled cells were washed with PBS. Fluorescence was determined using a flow cytometer (BD Biosciences LSRFortessa) and analyzed using FlowJo software (Tree Star, Inc, CA, USA). Live cells were first obtained by SSC and FSC, then dispersed cells were obtained by SSC‐A and SSC‐H. Finally, positive and negative cells in the experimental group were circled according to the fluorescence signal value of the control.

### Immunofluorescence Assay

The cells were rinsed with PBS and fixed with 4% paraformaldehyde at room temperature for 10 min and then permeabilized with 0.05% Triton X‐100 for 10 min. After 5% GSA blocking at room temperature for 1 h, the cells incubated sequentially with the specific primary antibodies overnight at 4 °C and fluorescent secondary antibodies in 5% bovine serum albumin for 1 h at 37 °C. Nuclei were stained with DAPI (Vector Laboratories, Italy). A confocal microscope (Nikon, Tokyo, Japan) was used to observe all stained slices.

### Co‐Immunoprecipitation

Protein samples were lysed in NETN150 lysis buffer. The lysates were quantified and incubated with indicated antibody overnight at 4 °C with rotation. In all, Protein A/G plus agarose beads (Santa Cruz, USA) were added to cell lysates and incubated for 2 h at 4 °C with rotation. The immunoprecipitants were collected by centrifugation and washed with NETN150 buffer 3 times. Immunoprecipitated proteins were resolved by SDS‐PAGE.

### Mass Spectrometry

HeLa 229 cells were lysed in NETN150 lysis buffer with protease inhibitor cocktails and phosphatase inhibitor cocktails (Sigma‐Aldrich, USA). The lysates were immunoprecipitated using an anti‐MUC1‐C antibody or IgG agarose overnight at 4 °C with rotation. Then, protein A/G plus agarose beads were added to cell lysates and incubated for 2 h at 4 °C with rotation. The beads were washed 5 times. Immunoprecipitation proteins were separated by SDS‐PAGE and stained with Coomassie blue. The band excised from the gel was subjected to reduction, carbamidomethylation, and tryptic digestion. Peptide sequences were determined by mass spectrometry using an Orbitrap Fusion LUMOS mass spectrometer (Thermo Scientific, USA) connected to an Easy‐nLC 1200 via an Easy Spray (Thermo Scientific, USA).

### GST Pull‐Down Assay

The plasmids for GST–PP2A‐Aα, ‐B55α, ‐B56α, and ‐Cα were transfected into BL21(DE3) (Weidi, China). The fusion proteins were prepared. MUC1–CD^WT^ or MUC1–CD^AQA^ protein and GST fusion protein were immobilized glutathione agarose and equilibrated before being incubated overnight at 4 °C with gentle rocking motion followed by washing 3 times with NETN150 buffer, the supernatant was discarded. The bound proteins were resolved by SDS‐PAGE.

### Cell Viability Assay

Cells were seeded in 96‐well plates and cell viability was determined using the cell counting kit 8 (CCK‐8) (NCM, China) according to the manufacturer's protocol. CCK‐8 was added and incubated for an additional 2 h, then the absorbance at the wavelength of 450 nm was measured by Multiskan FC (Thermo Scientific, USA) at the same time every day.

### PP2A Assay

As per the manufacturer's protocol, PP2A activity was measured using PP2A Immunoprecipitation Phosphatase Assay Kit (EMD Millipore). For each treatment condition, PP2A subunit C was immunoprecipitated. PP2A substrate threonine phosphopeptide was incubated with the eluate obtained by PP2A‐C immunoprecipitation. The amount of phosphate released as a result of the dephosphorylation of threonine phosphopeptide was measured and quantified with a malachite green solution.

### Xenograft Experiments

Animal protocols were in accordance with the Shanghai Medical Experimental Animal Care Guidelines. Research was approved by the Institutional Animal Care and Use Committee of the Shanghai Jiao Tong University School of Medicine. Female 6–8 weeks old athymic nude mice (BALB/c‐nu/nu) were injected with indicated cells as described previously. Treatment was started once the size of the xenograft reached 4 mm in diameter. Investigators were blinded to the group allocation. Tumor growth was monitored by a caliper ruler every 3 days. The volume was calculated according to the formula: *V* = 0.52 × length × width.

### Human SCLC Cancer Specimens and Immunohistochemistry

Patients who underwent small cell lung cancer surgery at the Shanghai Chest Hospital were randomly selected. Slides were reviewed and blocks were identified based on the presence of adequate tumors and the representative nature of the overall tumor. The age of the patients was 50–70 years old, and after the operation, all cases were confirmed by pathology. Immunohistochemistry analysis was performed as previously described.^[^ ^34^ ^]^ The tissue images were captured on the microscope (ZEISS, Jena, Germany). This study was approved by the Ethics Committee of the Shanghai Chest Hospital and each patient signed an informed consent form.

### Statistical Analysis

Statistical analyses were conducted using GraphPad Prism v8 (GraphPad Software) with one‐way ANOVA for single‐variable comparisons and two‐way ANOVA for multifactorial experimental designs. All data were presented as means ± standard deviation (SD) and represented three independent experiments. N.S. stood for not significant; * *p* < 0.05; ** *p* < 0.01; *** *p* < 0.001; and **** *p* < 0.0001.

## Conflict of Interest

The authors declare no conflict of interest.

## Supporting information



Supporting Information

## Data Availability

Data sharing is not applicable to this article as no new data were created or analyzed in this study.
