# Supplementary material for: PP2A Promotes the Symmetric Division of MUC1‐Dominant Cancer Stem‐Like Cells in Small Cell Lung Cancer
Source: Adv Sci (Weinh). 2025 May 11;12(25):2503545. doi: 10.1002/advs.202503545 (PMC12224998; doi:10.1002/advs.202503545)
Supplement: Supplementary file 1 — Supporting Information [file ADVS-12-2503545-s001.docx]

**Supporting Information**

**
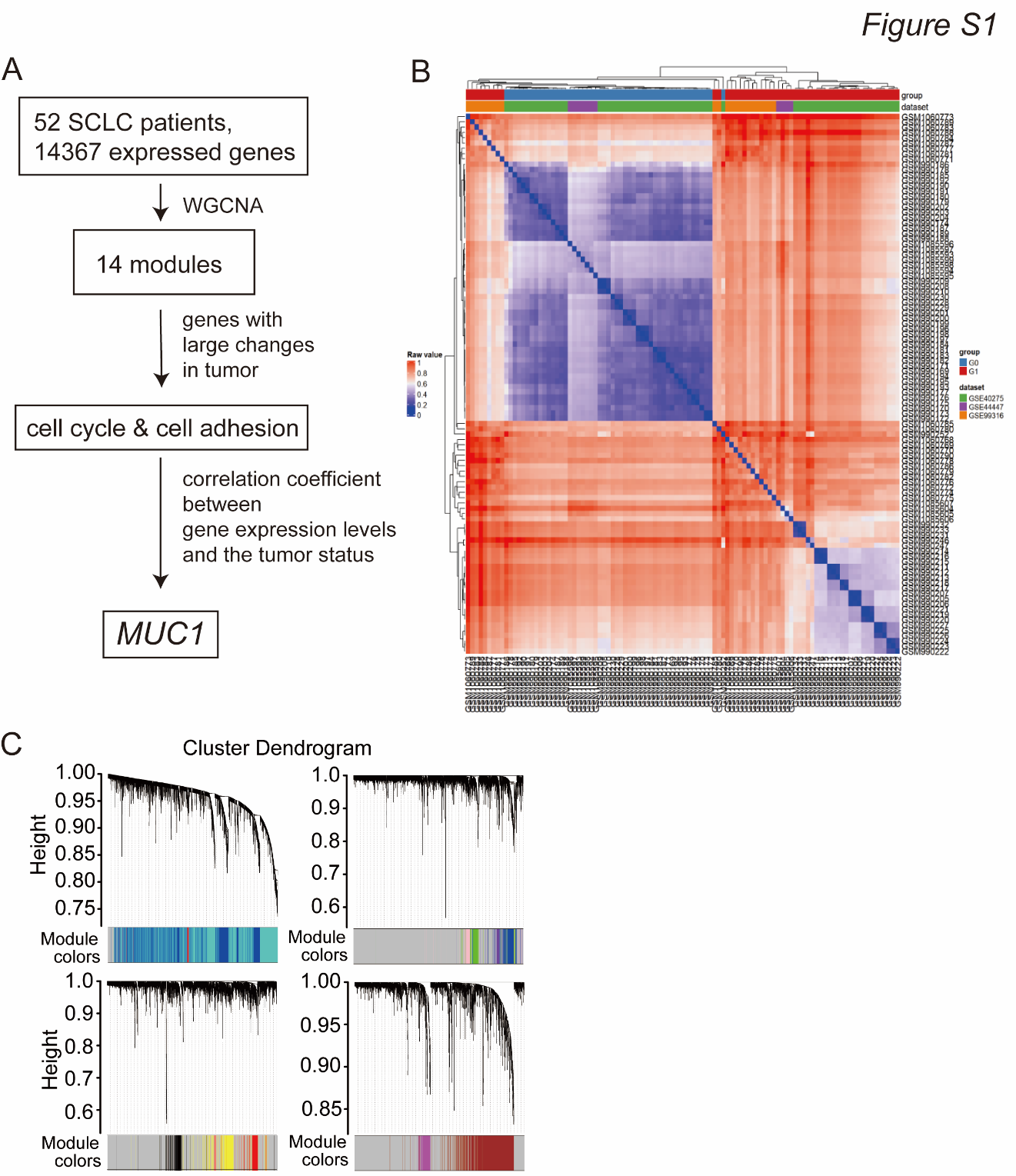
**

**Figure S1. SCLC patient information and MUC1 expression.**

(A) Flowchart for transcriptomics analysis of SCLC patient samples from the GSE database;

(B) Gene expression information of 52 SCLC samples versus 50 normal tissue samples, data from the TCGA database;

(C) WGCNA analysis of gene modules with changes in SCLC tissues.

**
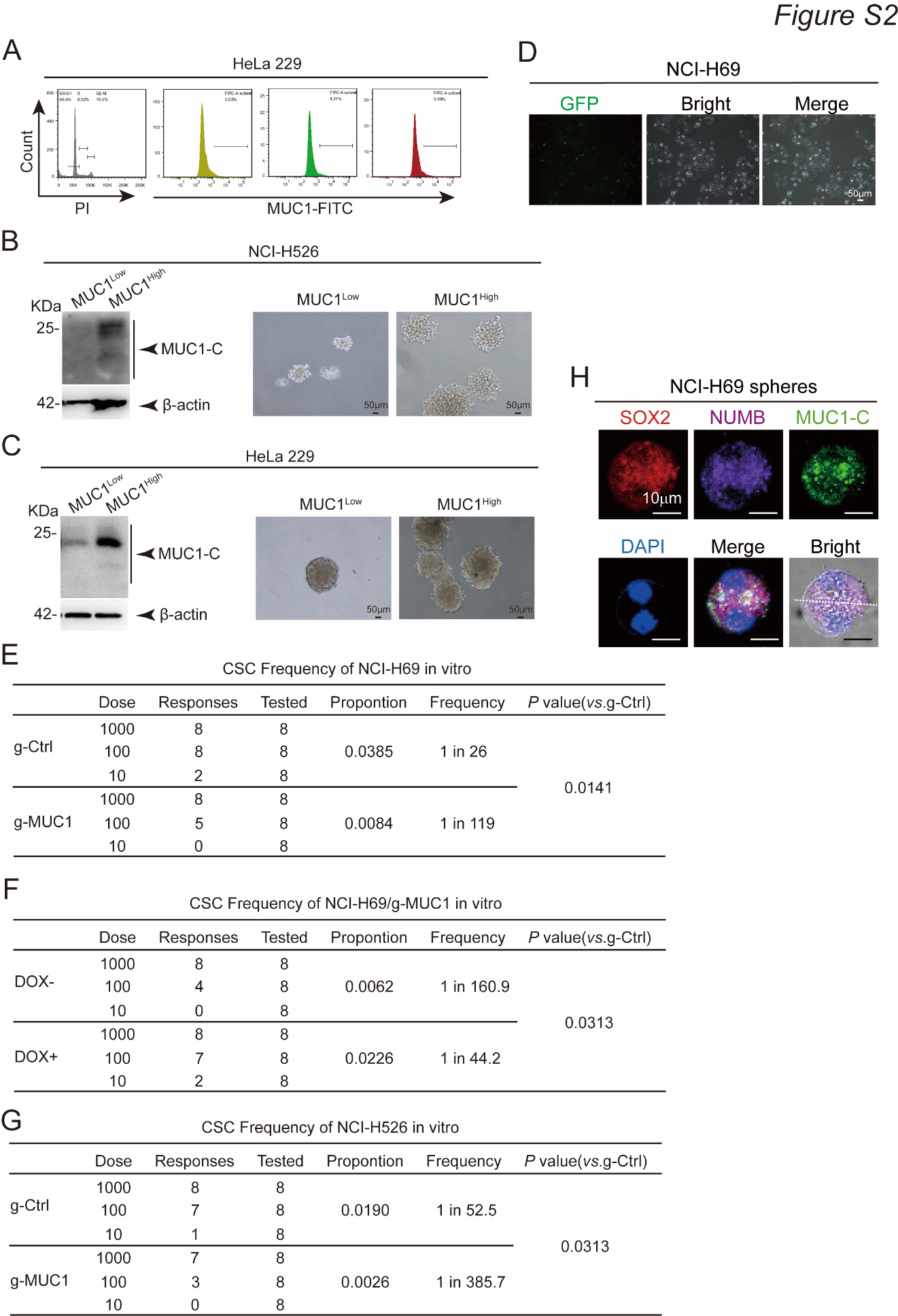
**

**Figure S2. Expression of MUC1 at different times and effects on sphere forming and stem cell frequency.**

(A) FCM on protein level of MUC1 in HeLa 229 cells at different periods;

(B–C) Detection of the effect of MUC1 expression on spheroidogenic ability in NCI-H526 (B) and HeLa 229 cells (C). Scale bars: 50 μm;

(D) Detection of the CRISPR knock-in efficiency of MUC1-GFP. Scale bars: 50 μm;

(E) *In vitro* stem-like cell frequency of NCI-H69 cells before and after MUC1 knockout were detected by ELDA;

(F) *In vitro* stem-like cell frequency of NCI-H69/g-MUC1 cells after induced expression of MUC1 were detected by ELDA;

(G) *In vitro* stem-like cell frequency of NCI-H526 cells before and after MUC1 knockout were detected by ELDA.

(H) Sphere-forming experiments were performed using NCI-H69 cells, enriched for cancer stem-like cells; spheres were digested into individual cells and continued to be cultured in sphere-forming medium while being treated with 25μM ovalbumin inhibitor Blebbistatin, and immunofluorescence assay was performed after 48h. The expression and distribution of SOX2, NUMB and MUC1 in the cells were observed.Scale bars: 10μm.


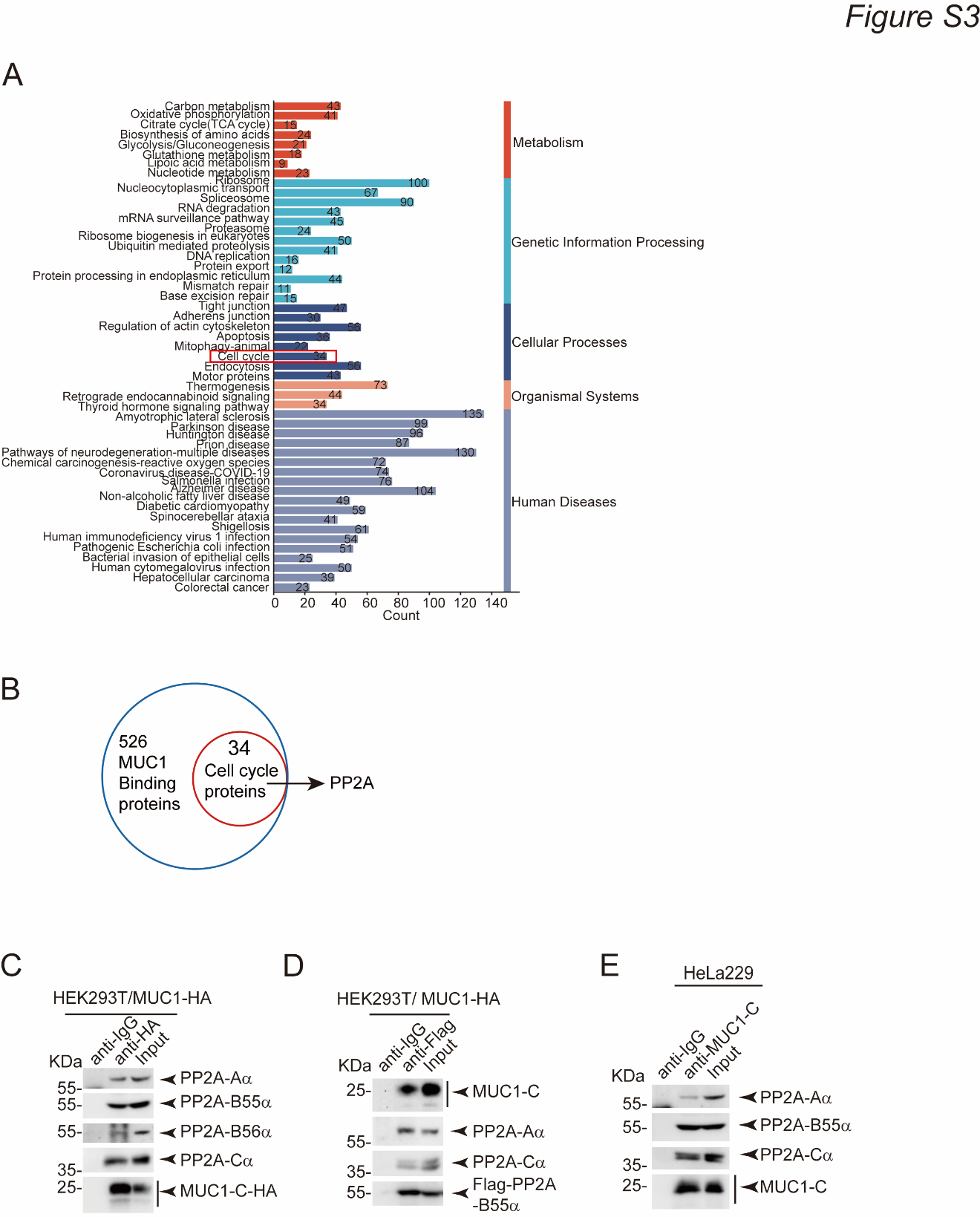


**Figure S3. MUC1 interacts with PP2A via CQC motif.**

(A) 526 proteins obtained by mass spectrometry were subjected to KEGG analysis;

(B) Mass spectrometry analysis revealed PP2A;

(C) MUC1 with HA tag at the C-terminus was transfected in HEK293T cells for 48 h, and cells were collected to perform co-IP with anti-HA antibody, western blot was detected the indicated proteins;

(D) MUC1 labeled with HA at the C-terminus and PP2A-B55α labeled with Flag were transfected into HEK293T cells for 48h, and cells were collected to perform co-IP with anti-Flag antibody, western blot was detected the indicated proteins;

(E) Co-IP was performed in HeLa 229 cells with anti-MUC1-C antibody, western blot was used to detect indicated proteins.


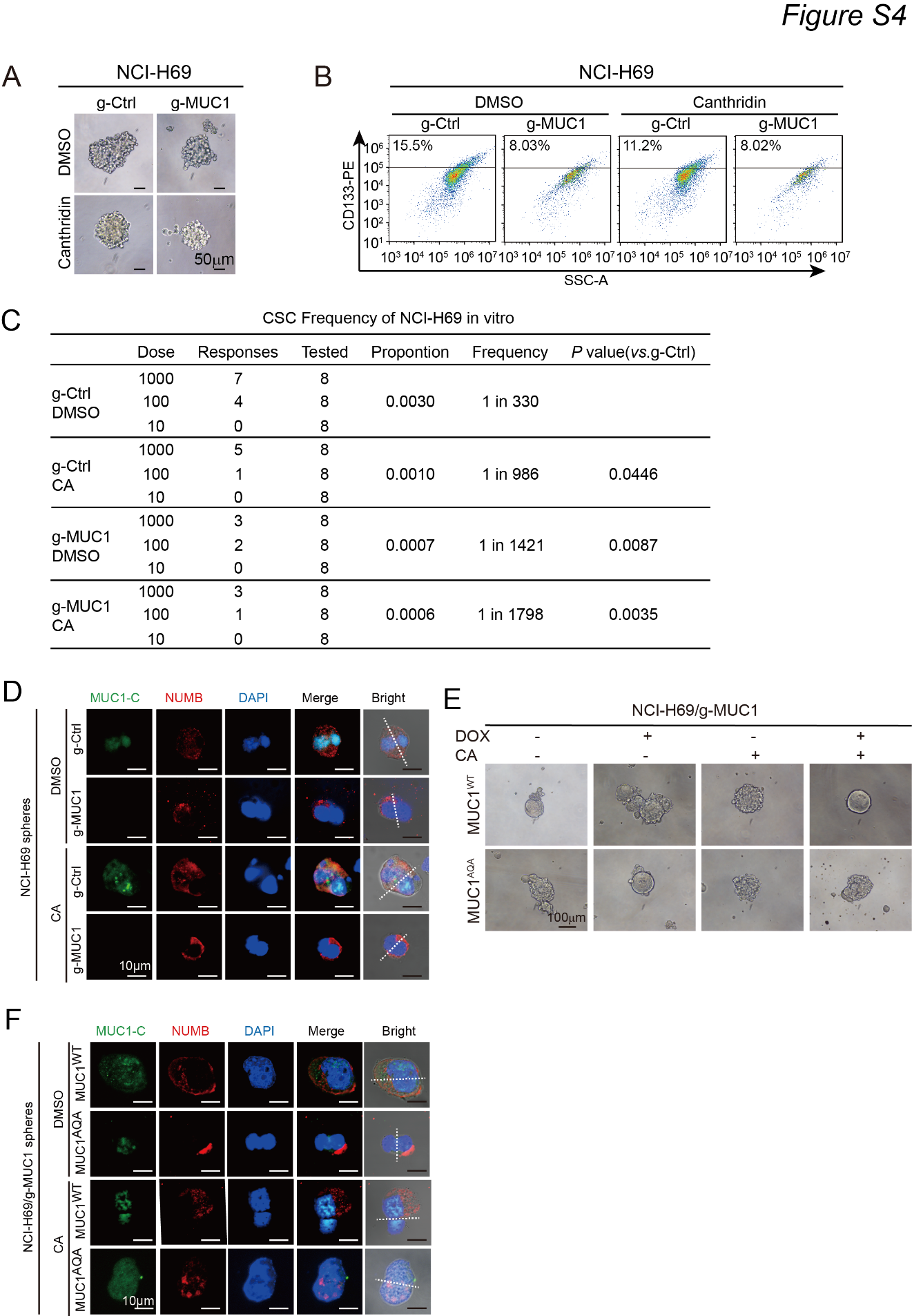


**Figure S4. Inhibition of PP2A suppresses symmetric division and tumor stemness.**

(A) NCI-H69/g-Ctrl and NCI-H69/g-MUC1 cells were treated with DMSO and 2 μM CA, respectively, and subjected to sphere-forming assay. Scale bars: 50 μm;

(B) NCI-H69/g-Ctrl and NCI-H69/g-MUC1 cells treated with DMSO and 2 μM CA for 48h, respectively, labeled with CD133-PE, and the proportion of CD133^+^ cells was detected by FCM assay;

(C) CSLCs frequencies of NCI-H69/g-Ctrl cells and NCI-H69/g-MUC1 cells upon CA treatment were detected by ELDA;

(D) CSLCs were enriched by sphere-forming assay using NCI-H69/g-Ctrl and NCI-H69/g-MUC1 cells, and then digested into individual cells and continued to be cultured with sphere-forming medium, and then cells were treated with DMSO and 2 μM Canthridin, respectively. After threated with 25 μM Blebbistatin for 48 h, cells were collected to perform IF assay to observe the distribution of NUMB and MUC1 in the cells. Scale bars: 10 μm;

(E) Expression of MUC1^WT^ and MUC1^AQA^ mutants were induced by DOX in NCI-H69/g-MUC1 cells. Sphere-forming assay was performed. Cells were treated with DMSO and 2 μM CA, respectively. Scale bars: 100 μm;

(F)Expression of MUC1^WT^ and MUC1^AQA^ was induced in NCI-H69/g-MUC1 cells, followed by sphere-forming assay to enrich cancer stem-like cells, and the spheres were separated into individual cells, which continued to be cultured with sphere-forming medium, and the cells were treated with DMSO and 2 μM Canthridin, respectively, 25 μM Blebbistatin was added to all the cells, and after 48 h, IF assay was subjected to observe the distribution of NUMB and MUC1 in the cells. Scale bars: 50 μm.


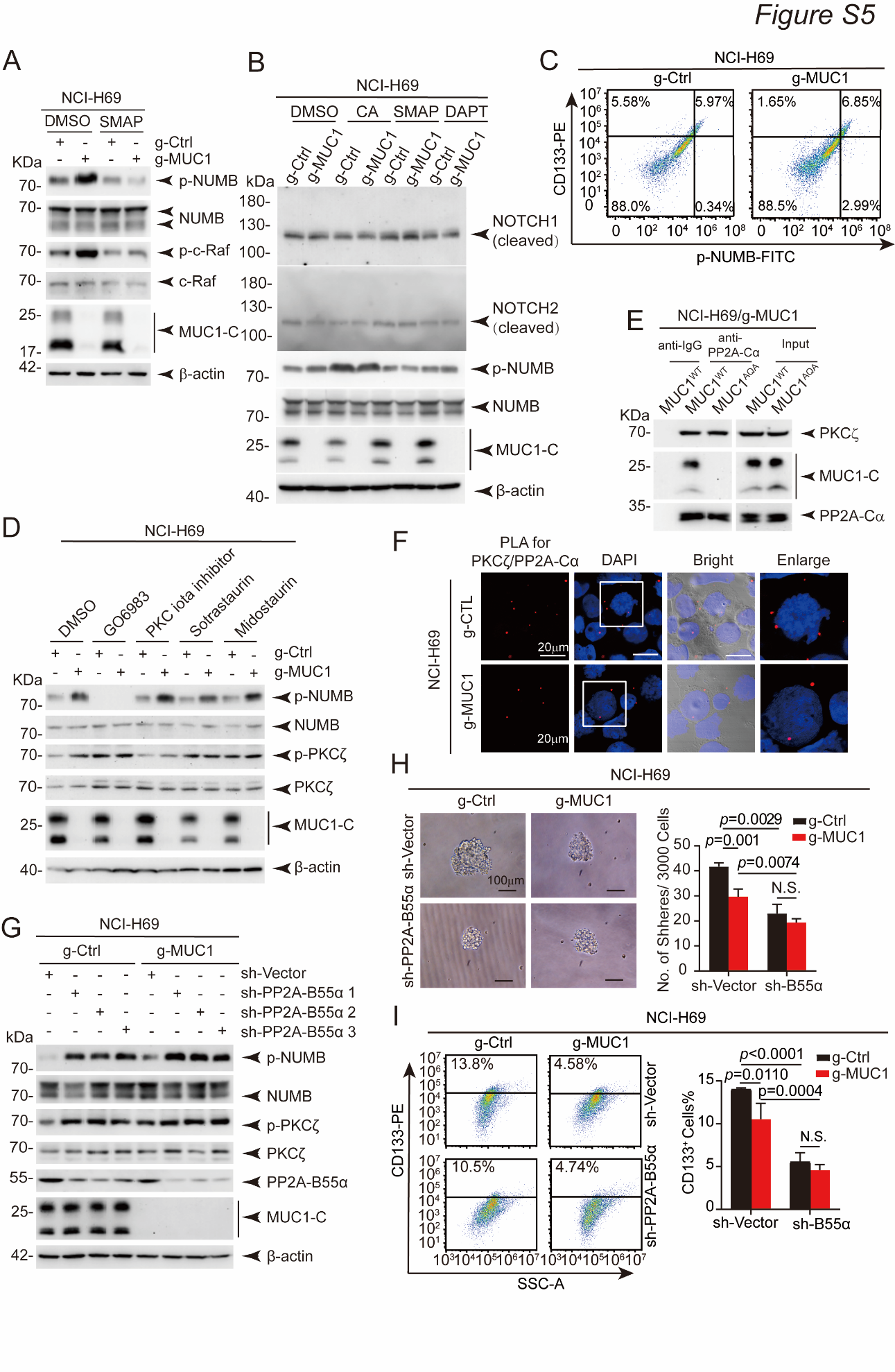


**Figure S5. MUC1-PP2A decreases phosphorylation of PKCζ and of NUMB, and improves tumor stemness.**

(A) NCI-H69/g-Ctrl and NCI-H69/g-MUC1 cells were treated with DMSO and 5μM SMAP for 48h, respectively. Cells were collected, and subjected to western blot to detected the indicated proteins with β-actin as an internal reference;

(B) NCI-H69/g-Ctrl and NCI-H69/g-MUC1 cells were treated with DMSO, 2 μM Canthridin, 5 μM SMAP and 5 μM DAPT for 48 h, respectively. Cells were collected, and subjected to western blot to detected the indicated proteins with β-actin as an internal reference;

(C) NCI-H69/g-Ctrl and NCI-H69/g-MUC1 cells were labeled with both CD133 and p-NUMB, and the proportion of CD133 and p-NUMB positivity was detected by FCM assay;

(D) NCI-H69/g-Ctrl and NCI-H69/g-MUC1 cells were treated with 5 μM GO-203, 5 μM PKC-iota inhibitor, 5 μM Sotrastaurin and 5 μM Midostaurin for 48 h, respectively. Cells were collected, and subjected to western blot to detected the indicated proteins with β-actin as an internal reference. GO6983 is an inhibitor of PKCζ, PKC-iota inhibitor 1 is an inhibitor of PKCι, Sotrastaurin is an inhibitor of PKCα/β/θ/η/δ/ε, and Midostaurin is an inhibitor of PKCα/β/γ;

(E) Expression of MUC1^WT^ and MUC1^AQA^ was induced in NCI-H69/g-MUC1 cells with DOX, respectively, and cells were collected for co-IP with anti-PP2A-Cα antibody, western blot was used to detect indicated proteins;

(F) Co-localization of PP2A-Cα and PKCζ was detected by proximity ligation assay (PLA). Scale bars: 20 μm;

(G) NCI-H69/g-Ctrl and NCI-H69/g-MUC1 cells with empty vector or PP2A-B55α knockdown were collected, and subjected to western blot to detected the indicated proteins with β-actin as an internal reference;

(H) NCI-H69/g-Ctrl and NCI-H69/g-MUC1 cells with empty vector or PP2A-B55α knockdown were collected, and subjected to sphere-forming assay. Scale bars: 100 μm. The data are presented as the mean ± SD from three independent experiments;

(I) NCI-H69/g-Ctrl and NCI-H69/g-MUC1 cells with empty vector or PP2A-B55α knockdown were collected, and subjected to FCM assay. The data are presented as the mean ± SD from three independent experiments.


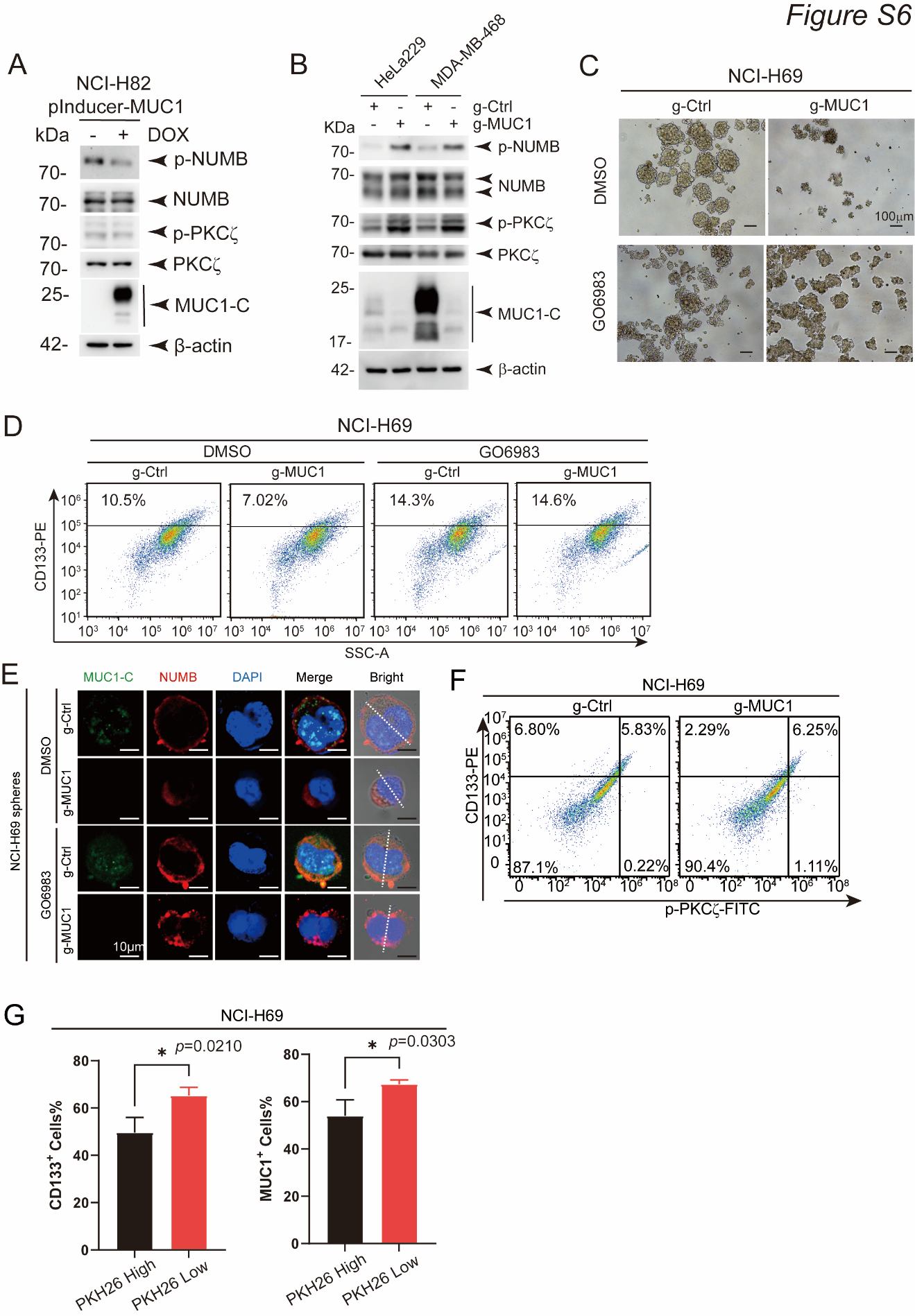


**Figure S6. Inhibition of PKCζ activity promotes symmetric division and tumor stemness.**

(A) Expression of MUC1 was induced by DOX in NCI-H82 cells, respectively. Cells were collected to detect the indicated proteins by western blot with β-actin as an internal reference.

(B) HeLa229/g-Ctrl and g-MUC1, MDA-MB-468/g-Ctrl and g-MUC1 cells were collected, and subjected to western blot to detected the indicated proteins with β-actin as an internal reference;

(C) NCI-H69/g-Ctrl and NCI-H69/g-MUC1 cells were treated with DMSO and 5 μM GO6983, respectively, and subjected to sphere-forming assay. Scale bars: 100μm;

(D) NCI-H69/g-Ctrl and NCI-H69/g-MUC1 cells were treated with DMSO and 5 μM GO6983 for 48 h, respectively, labeled with CD133-PE, and the proportion of CD133^+^ cells was detected by FCM assay;

(E) CSLCs were enriched by sphere-forming assay using NCI-H69/g-Ctrl and NCI-H69/g-MUC1 cells, and then digested into individual cells and continued to be cultured with sphere-forming medium; and then cells were treated with DMSO and 5 μM GO6983, respectively. After treated with 25 μM Blebbistatin for 48 h, cells were collected to perform IF assay to observe the distribution of NUMB and MUC1 in the cells. Scale bars: 10 μm;

(F) NCI-H69/g-Ctrl and NCI-H69/g-MUC1 cells were labeled with both CD133 and p-PKCζ, and the proportion of CD133 and p-PKCζ positivity was detected by FCM assay;

(G) 7 days after PKH26 labeling, NCI-H69 cells were flow-sorted to obtain two fractions of cells with high PKH26 and low PKH26, and then labeled with CD133 and MUC1 at the same time. The proportion of CD133^+^ cells or MUC1^+^ were detected by FCM assay. The data are presented as the mean ± SD from three independent experiments.


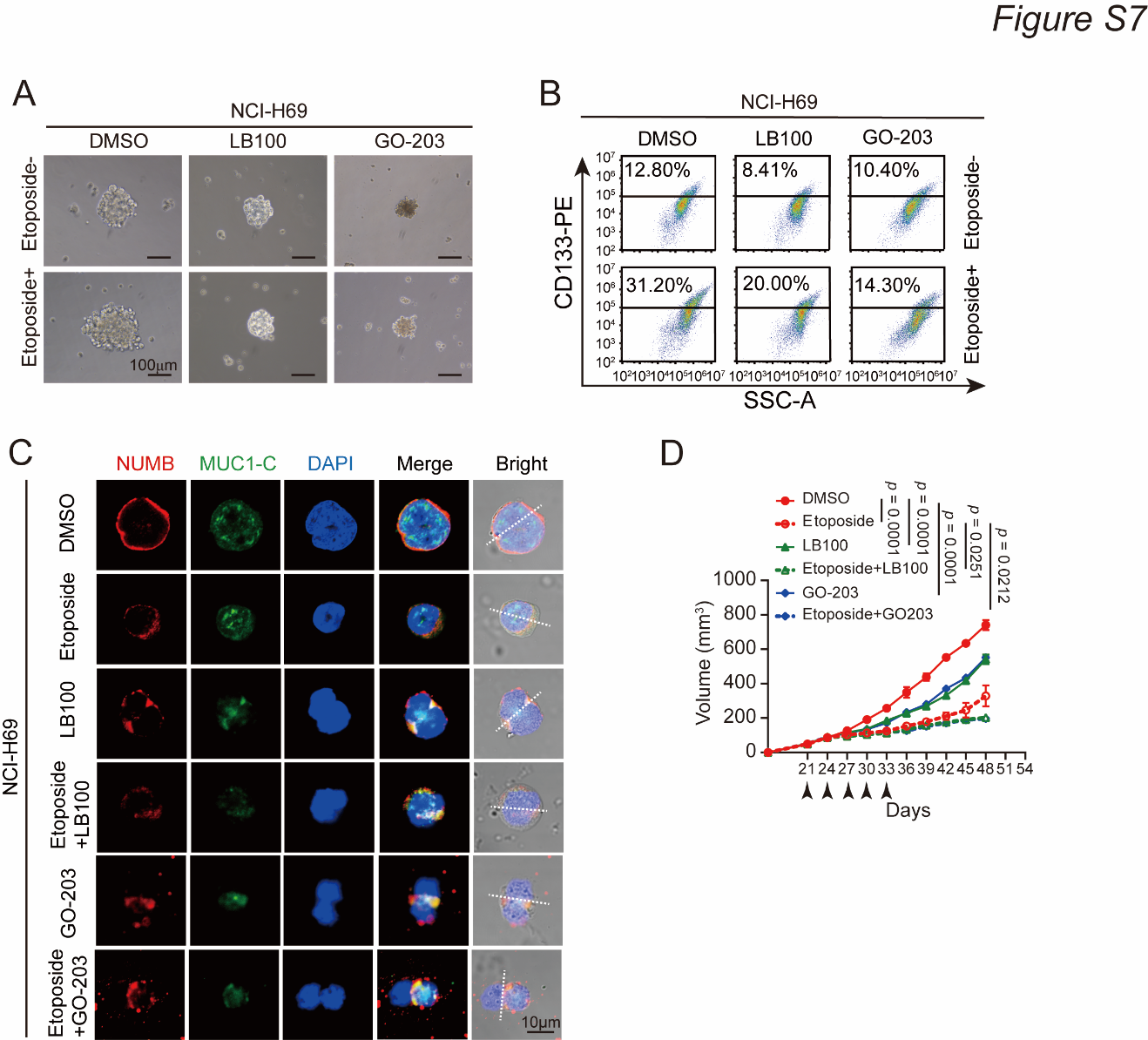


**Figure S7. Targeting MUC1-PP2A inhibits symmetric division and tumor stemness.**

(A) NCI-H69 cells were treated with DMSO, 5 μM etoposide, 10 μM LB100, etoposide+LB100, 5 μM GO-203, and etoposide+GO-203, respectively, and subjected to sphere-forming assay. Scale bars: 100 μm;

(B) NCI-H69 cells were treated with DMSO, 5 μM etoposide, 10 μM LB100, etoposide+LB100, 5 μM GO-203, and etoposide+GO-203 for 48 h, respectively, labeled with CD133-PE. The proportion of CD133^+^ cells was detected by FCM assay;

(C) CSLCs were enriched by sphere-forming assay using NCI-H69 cells, and then digested into individual cells and continued to be cultured with sphere-forming medium, and then cells were treated with DMSO, 5 μM etoposide, 10 μM LB100, etoposide+LB100, 5 μM GO-203, and etoposide+GO-203, respectively. After treated with 25 μM Blebbistatin for 48 h, cells were collected to perform IF assay to observe the distribution of NUMB and MUC1 in the cells. Scale bars: 10μm;

(D) NCI-H69 cells were subjected to mouse xenograft assays. Mice were treated with DMSO, etoposide (20mg/kg, once every three days), LB100 (2.5mg/kg, every day), combination of etoposide (20mg/kg, once every three days) and LB100 (2.5mg/kg, every day), GO-203 (15mg/kg, every day), or combination of etoposide (20mg/kg, once every three days) and GO-203 (15mg/kg, every day) for 15 days. Tumor sizes were monitored. The data are presented as the mean ± SD, *n*=6.

**
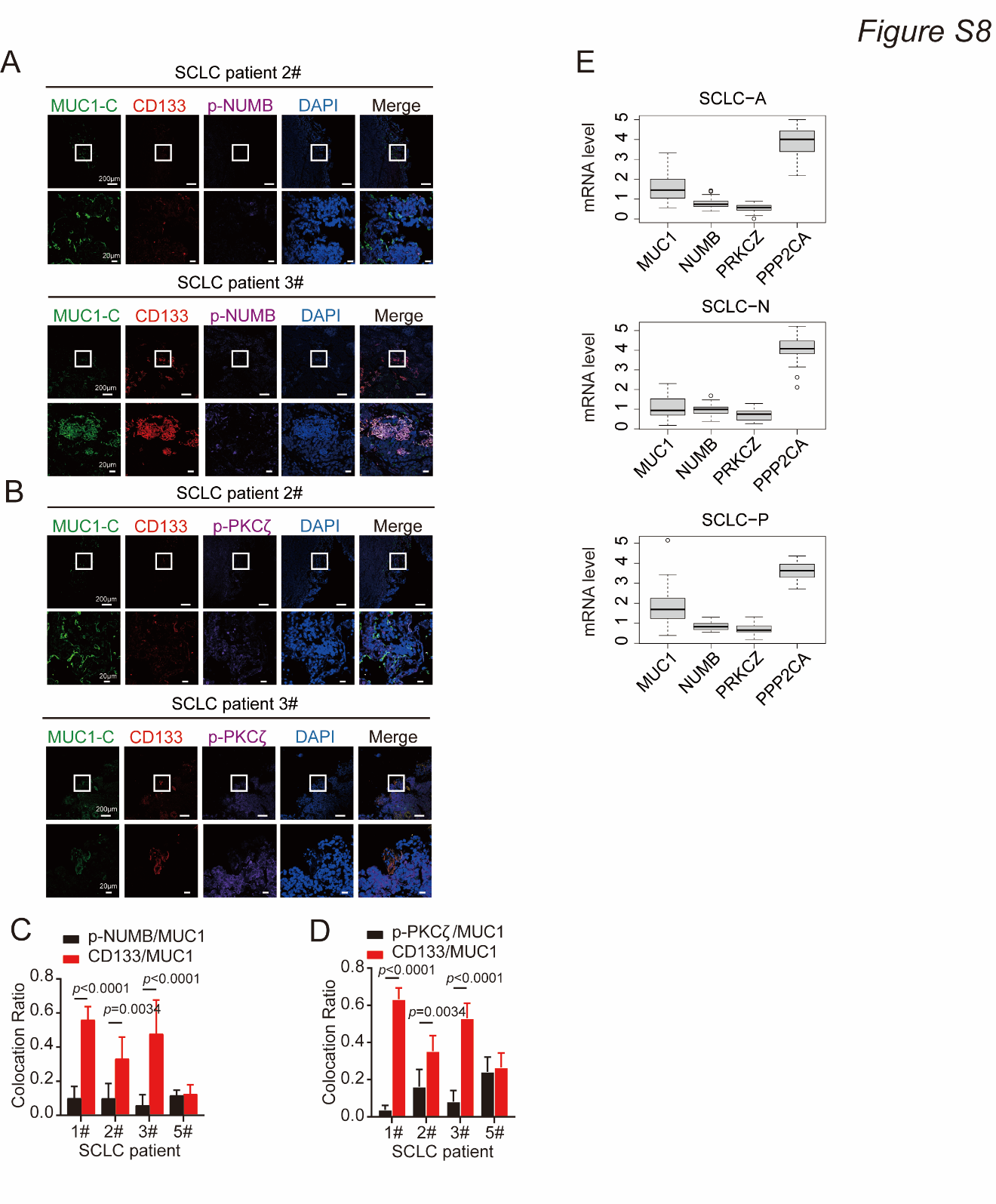
**

**Figure S8. MUC1-PP2A signaling axis is present in different subtypes of SCLC.**

(A-D) Co-localization of MUC1-C and p-NUMB (A), MUC1-C and CD133, MUC1-C and p-PKCζ (B) in tissues derived from dissected tumors of SCLC patients were detected by IF assay, Scale bars: 200μm. Statistically analyze the co-location ratio (C-D). The data are presented as the mean ± SD, *n*=5;

(E) Transcript levels of MUC1, NUMB, PKCζ and PP2A-Cα in different subtypes of SCLC;
